# Data analysis of the unsteadily accelerating GPS and seismic records at Campi Flegrei caldera from 2000 to 2020

**DOI:** 10.1038/s41598-022-23628-5

**Published:** 2022-11-10

**Authors:** Andrea Bevilacqua, Prospero De Martino, Flora Giudicepietro, Patrizia Ricciolino, Abani Patra, E. Bruce Pitman, Marcus Bursik, Barry Voight, Franco Flandoli, Giovanni Macedonio, Augusto Neri

**Affiliations:** 1 Istituto Nazionale di Geofisica e Vulcanologia, Sezione di Pisa, Pisa, Italy; 2 Istituto Nazionale di Geofisica e Vulcanologia, Osservatorio Vesuviano, Napoli, Italy; 3 Department of Mathematics, Tufts University, Medford, MA USA; 4 Department of Materials Design and Innovation, University at Buffalo, Buffalo, NY USA; 5 Department of Earth Sciences, University at Buffalo, Buffalo, NY USA; 6 Department of Geology, Pennsylvania State University, State College, PA USA; 7 Scuola Normale Superiore di Pisa, Pisa, Italy

**Keywords:** Volcanology, Geophysics

## Abstract

Ongoing resurgence affects Campi Flegrei caldera (Italy) via bradyseism, i.e. a series of ground deformation episodes accompanied by increases in shallow seismicity. In this study, we perform a mathematical analysis of the GPS and seismic data in the instrumental catalogs from 2000 to 2020, and a comparison of them to the preceding data from 1983 to 1999. We clearly identify and characterize two overlying trends, i.e. a decennial-like acceleration and cyclic oscillations with various periods. In particular, we show that all the signals have been accelerating since 2005, and 90–97% of their increase has occurred since 2011, 40–80% since 2018. Nevertheless, the seismic and ground deformation signals evolved differently—the seismic count increased faster than the GPS data since 2011, and even more so since 2015, growing faster than an exponential function The ground deformation has a linearized rate slope, i.e. acceleration, of 0.6 cm/yr^2^ and 0.3 cm/yr^2^ from 2000 to 2020, respectively for the vertical (RITE GPS) and the horizontal (ACAE GPS) components. In addition, all annual rates show alternating speed-ups and slow-downs, consistent between the signals. We find seven major rate maxima since 2000, one every 2.8–3.5 years, with secondary maxima at fractions of the intervals. A cycle with longer period of 6.5–9 years is also identified. Finally, we apply the probabilistic failure forecast method, a nonlinear regression that calculates the theoretical time limit of the signals going to infinity (interpreted here as a critical state potentially reached by the volcano), conditional on the continuation of the observed nonlinear accelerations. Since 2000, we perform a retrospective analysis of the temporal evolution of these forecasts which highlight the periods of more intense acceleration. The failure forecast method applied on the seismic count from 2001 to 2020 produces upper time limits of [0, 3, 11] years (corresponding to the 5th, 50th and 95th percentiles, respectively), significantly shorter than those based on the GPS data, e.g. [0, 6, 21] years. Such estimates, only valid under the model assumption of continuation of the ongoing decennial-like acceleration, warn to keep the guard up on the future evolution of Campi Flegrei caldera.

## Introduction

**Figure 1:**
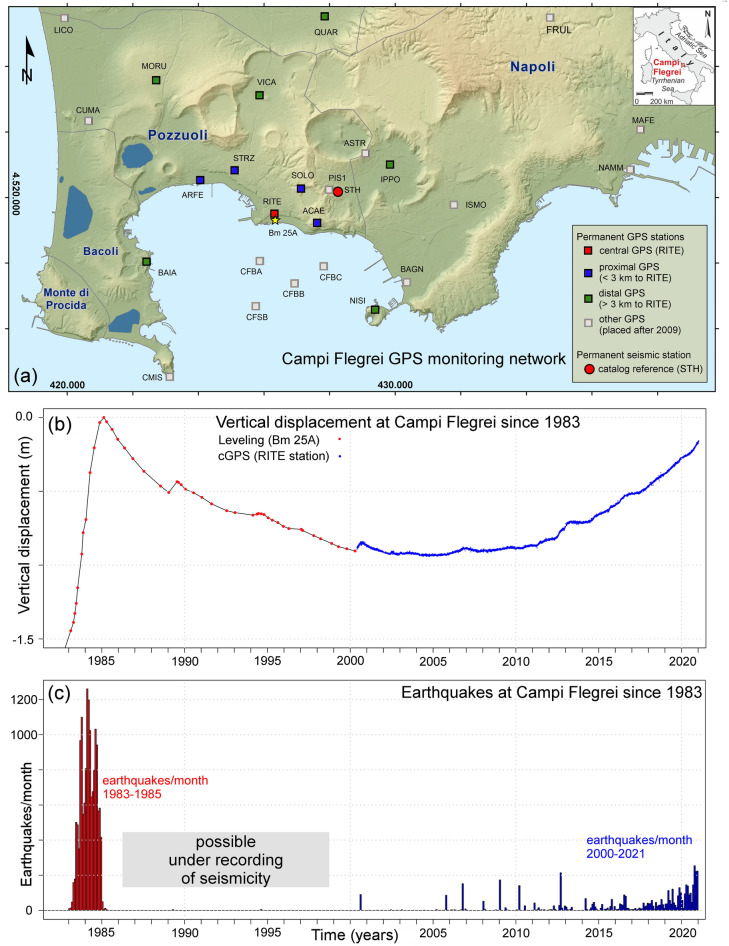
(**a**) Campi Flegrei caldera GPS monitoring network (small squares, 21 permanent stations + 4 buoys offshore)^27^. Background map modified from the INGV periodic bulletin of Campi Flegrei^8^. The 11 analyzed stations are marked with colors: RITE station is red, 4 proximal stations are blue, 6 distal stations are green. Other GPS stations and buoys are gray. The seismic catalog reference station STH is a red circle. (**b**) Overview of the ground displacement at Campi Flegrei in 1983–2020. The data merge leveling data, collected at benchmark 25A, and the RITE GPS. (**c**) Overview of the earthquakes recorded at Campi Flegrei in 1983–2020. In (**b**,**c**) the data collected in 2000–2020 are marked in blue. Data modified from the INGV periodic bulletin of Campi Flegrei (http://www.ov.ingv.it).

The Campi Flegrei caldera (CFc, Fig. 1a) is a restless, complex structure, and the site of widespread hydrothermal phenomena. An ongoing resurgence affects the caldera floor through bradyseism, i.e. a series of short-term episodes documented since the 1950s, and accompanied by increases in shallow seismicity^51,52,54,89,97^. CFc hosts a densely inhabited urban area home to about 350,000 people, including the western neighborhoods of the city of Naples^37,82,90^. Hazard assessment is thus particularly critical^15,22,86,103^, and the interaction of the active magmatic and hydrothermal systems complicates the interpretation of monitoring data^26^.

In general, an impending eruption of CFc would likely be associated with a substantial acceleration in the unrest signals^41,74,96,101,102^. Nevertheless, during prolonged unrest, a caldera can produce intense geodetic, seismic, and geochemical effects not followed by an eruption, and, at the same time, the final precursors before the eruption may be weaker than those registered previously^1,87,88^. Therefore, great uncertainty characterizes the temporal evolution of the CFc system. Some isotope-geochemical investigations of the products of the most recent eruptions, and the experience with “analogue” volcanoes have suggested that clear evidence of an upcoming eruption may become detectable only a few days/hours before the actual event^91,95^. However, the concept of analogue volcanoes still lacks of a clear definition, and the geochemical investigations on the magma transit from the chamber to the surface might produce unrealistic results if they do not focus on the first magma that reaches the surface. In fact, large-scale variations preceded the AD 1538 eruption for decades, with a final phase of a couple of months of strong seismicity and rapid uplift of the vent area occurring only a few days before eruption onset^59,60,69^. Moreover, the macroscopic pre-eruptive phenomena mentioned in the historical sources are only those that become visible when the magma is at very shallow level, probably in the order of hundreds of meters. In contrast, a modern dense instrumental network may be able to detect magma ascent in advance, i.e. from a few kilometers depth.

In this study, we tackle the problem of the mathematical analysis of the GPS and seismic data by considering the most complete instrumental catalogs of ground displacement and earthquakes in CFc, up to 31/12/2020^27,54,67^. The main target of our study is measuring and modeling the main features of the geophysical monitoring signals described in terms of temporal rates, Fourier analysis and differential modeling of nonlinear acceleration.

In “The ground displacement and seismic datasets collected in CFc” section, we present our two input datasets, e.g. the GPS and seismic catalogs, and introduce relevant scientific background. In “Data analysis of GPS and seismic catalogs” section, we measure and discuss the seismicity and uplift rates from 2000 to 2020, further detailing the properties of the signals collected in 2018–2020 and including an analysis of 1983–1985. In “Recurrent oscillations in 1985–2020” section, we highlight the main harmonics shared by earthquake count and ground deformation from 1985 to 2020, and we calculate the Fourier spectrum of the GPS measured displacement rate after subtracting a linear trend. In “The FFM tool and its probabilistic formulation” section, we focus on the differential modeling of the nonlinear acceleration of the signals. Thus, we introduce the theory of the failure forecast method (FFM)^116,117^ and its probabilistic enhancement (pFFM)^17^. We report on the example of nonlinear regression and stochastic extrapolation of the forecast of failure time made on 01/01/2021, and we perform a sensitivity analysis in terms of the type of data utilized, the length of the regression, and the time step in the signals’ rate calculation. Then, we describe the evolution of the FFM forecasts between 2000 and 2020, showing how the forecast changed through time. It is worth clarifying that the frequency analysis in “Recurrent oscillations in 1985–2020” section and the pFFM analysis in “The FFM tool and its probabilistic formulation” section are parallel and complementary approaches. In fact, they describe two overlying trends, i.e. cyclic oscillations and a decennial-like acceleration of data. In “Results application and interpretation” section, we briefly discuss possible physical interpretations of the results based on existing literature models and discuss the capabilities and limitations of our analysis as a tool for volcano monitoring and surveillance.

Results of the combined analyses allow us to clearly identify and characterize two overlying trends in the geophysical data, i.e. the decennial-like acceleration and cyclic oscillations with various periods (years/months). The decennial-like accelerating trend of the geophysical data appears as particularly relevant. We investigate it by evaluating its properties and the chance that the volcano reaches a critical state in the next decades, in case of its continuation with the same characteristics. We also speculate on its possible influence in amplifying the effects of new periodic ground oscillations.

## The ground displacement and seismic datasets collected in CFc

In this study, we analyze the GPS and seismic data recorded in recent years, i.e. decadal datasets of continuous instrumental measurements, data that is uncommon for calderas worldwide^27^.

### The GPS datasets of CFc from 2000 to 2020

The Neapolitan Volcanoes Continuous GPS network, operated by INGV-OV, comprises 21 continuous GPS stations operating on land in the Campi Flegrei area, plus 4 installed on buoys connected to the seafloor (Fig. 1a)^53,54^. The daily position time series of the 21 on land stations are included in De Martino et al.^54^. We focus our analysis on the 11 stations installed before 2009, thus having collected the longest time series.

Figure 1b provides an overview of the deformation history of CFc since 1983^51,100^. In the twentieth century CFc went through three major bradyseismic crises, in 1950–53, 1969–72 (both not shown in the figure) and in 1982–84, with a ground uplift of 0.75 m, 1.70 m and 1.85 m, respectively^10,11,24,49,78,89^. The maximum-recorded vertical difference between 1950 and 1985 is 3.75 m. About 20 years of overall subsidence took place from 1985 to 2005, producing a vertical difference of − 0.80 m. Afterwards, the Campi Flegrei caldera has been rising again, starting slowly^112^, but accelerating through the years^84^. The maximum vertical displacement in the central area almost reached 80 cm in 01/2021, nearly back to the uplift level of 1985^52,54^.

INGV placed the first continuous GPS stations in the Campi Flegrei area during the 2000 mini-uplift, i.e. a maximum vertical displacement of about 4 cm that briefly interrupted the subsidence ongoing at that time^25,62^. Then, after 2005 the RITE GPS station located at Rione Terra-Pozzuoli steadily recorded the largest uplift. Note that the inversion of the GPS data places the center of the bell about 500 m in the sea, SW of the Rione Terra^2,3^. The other GPS stations typically show a bell-shaped decrease in the vertical displacement from the caldera center outwards, and a radial symmetry in the horizontal displacements centered on Pozzuoli and with maxima located on a half-annulus of 2–3 km radius^18^.

### The STH seismic catalog of CFc from 2000 to 2020

The INGV-OV permanent seismic network currently consists of 40 digital stations and 5 analog seismic stations^67^. In this study we consider the 01/2000–12/2020 seismic catalog of Campi Flegrei that is routinely updated by the Osservatorio Vesuviano seismic laboratory^93^. The considered catalog contains 4845 earthquakes recorded from 2000 to 2020 at the STH reference station, installed near the Solfatara-Pisciarelli area (Fig. 1a). The completeness magnitude in this dataset is 0.2 to 0.4, and when the background seismic noise is particularly low, this station can record earthquakes with magnitude > − 0.2^67,109,110^.

Seismicity typically occurs in CFc during the uplifting phases, whereas it is almost absent during subsidence^77^. The higher the uplift rate, the stronger and more pronounced is the seismicity. The uplift of 1950–52 was not accompanied by felt seismicity^51^. The uplift of 1969–72 was accompanied by a moderate seismicity with a few felt earthquakes and hundreds of low magnitude events, but relatively larger earthquakes occurred in 1983–1984, with maximum magnitude of 4.0–4.2 (Fig. 1c)^4,30,57,89^.

**Figure 2:**
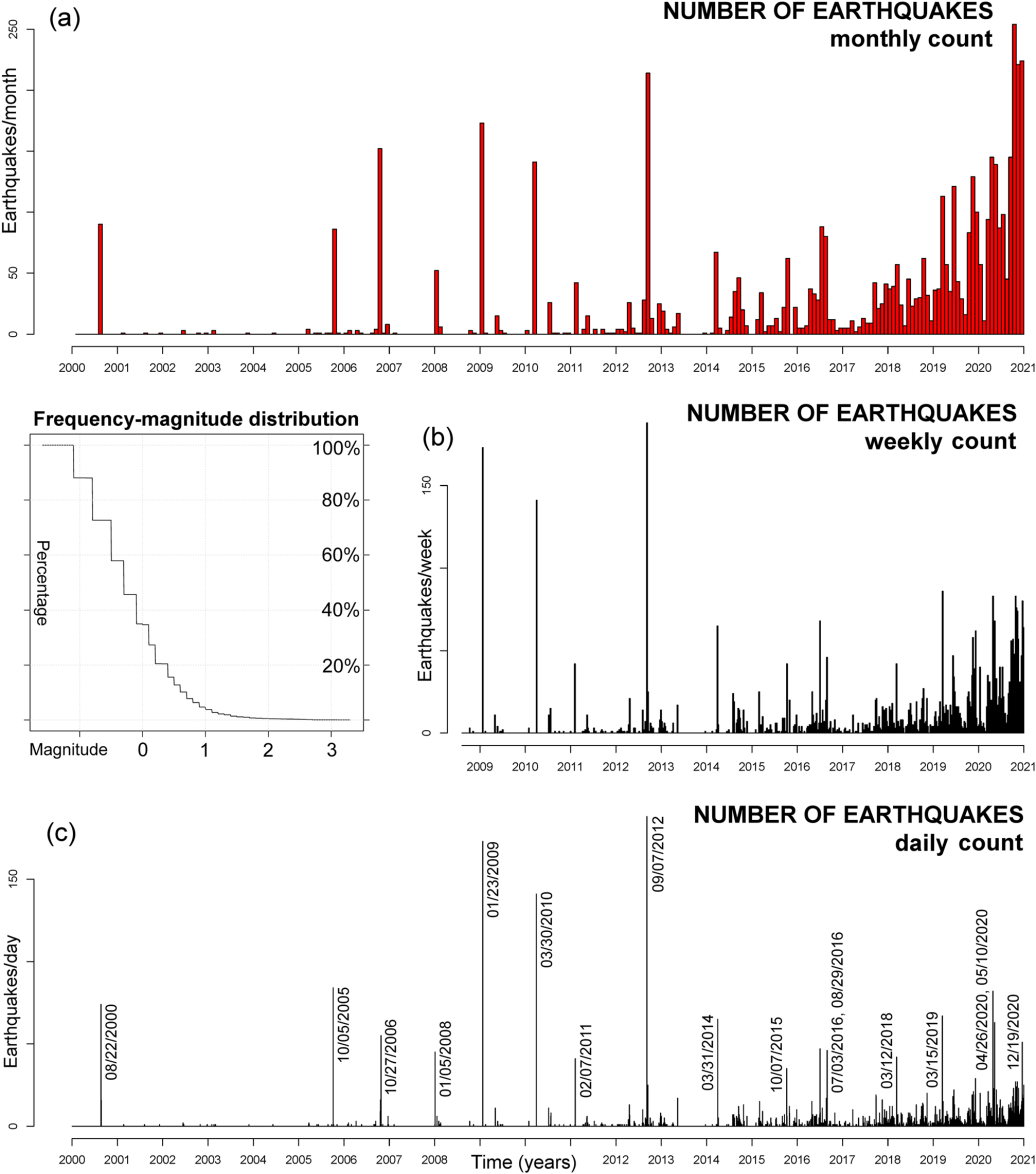
Overview of the earthquakes recorded at Campi Flegrei in 2000–2020. Plot (**a**) shows the monthly count, plot (**b**) the weekly count, plot (c) the daily count. In (**c**) the days with more than 30 events are labeled. The Frequency-magnitude distribution is shown on the left of (**b**). The cumulative number of earthquakes is shown in Fig. 3d.

Figure 2 shows that, at least since 2005, a gradual increase in seismicity has occurred in CFc. Until 2014, earthquakes were rare and occurred in swarms up to 100–200 events with low magnitudes, but afterwards the events became more frequent over time and the earthquakes not occurring in swarms significantly increased in magnitude. The Gutenberg-Richter b-value increased through time—from values smaller than 0.8 in 2014 to values close to 1.3 at the end of 2019^109^; this may reflect the increasing injection of magmatic fluids into the hydrothermal system and its pressurization^65,111^. Several of the seismic swarms in this time interval affected the Solfatara-Pisciarelli area^110^, where has been recorded the greatest increase in the rise of flux of fluids, however many other earthquakes occurred elsewhere, mostly concentrating in a 3 km radius from the center of the caldera^67^.

In the following analysis, we separate the swarms from the “background” seismicity and analyze both^42^. In fact, by removing the swarms we obtain a more regular time series, which nevertheless shows similar features to the entire dataset.

## Data analysis of GPS and seismic catalogs

For the sake of clarity, in each figure of this section, we only show a selection of examples of GPS and seismic data. Supporting information S1 includes all the data and analyses performed without exception. Namely, in this section we consider:

- The vertical ground displacement and the horizontal ground displacement, i.e. the length of the vector defined by the E-W and N-S spatial components.
- The cumulative count of the earthquakes recorded. Specifically, we counted all recorded events and then empirically excluded the swarms, i.e. by assuming no more than one event per day.
- The cumulative energy of the earthquakes and the Benioff strain release, i.e. 10^2.9 + 1.9Md^ and its square root, respectively^9,94^.

In the selected examples, we choose the RITE and ACAE GPS stations to represent the maximum collected vertical displacement and horizontal displacement components, respectively. In many cases, we focus on the description of ACAE, because these two signals share a very similar pattern. Since we analyze tens of years of data, we adopt a simplified definition of swarm, i.e. the occurrence of multiple seismic events in the same calendar day. Note that we also considered the dataset of all seismic events, which shares features with the dataset excluding swarms.

**Figure 3:**
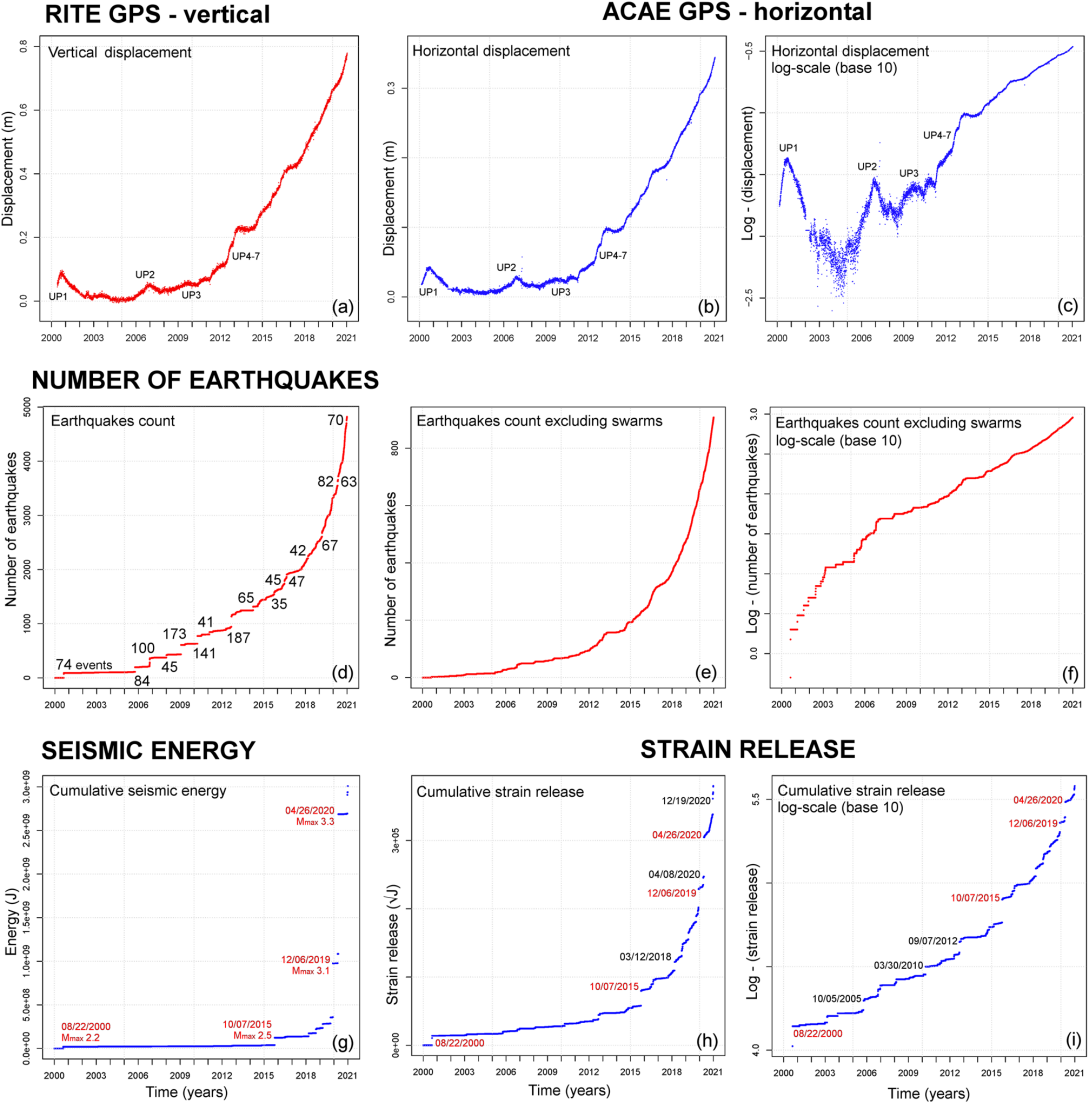
Examples of GPS and seismic data. Plots (**a**–**c**) show the ground displacement: plot (**a**) is the vertical displacement recorded at RITE GPS station; plots (**b**, **c**) are the horizontal displacement recorded at ACAE GPS station. Plots (**d**–**f**) show the cumulative number of seismic events recorded: plot (**d**) includes all the events in the catalog, and we labeled the size of the swarms of > 30 events; plots (**e**–**f**) exclude the swarms, i.e. by counting no more than one seismic event per day. Plots (g-i) show the cumulative seismic energy (**g**) and strain release (**h**–**i**) estimation. Most energetic earthquakes are labeled, in red the four events of 08/22/2000, 10/07/2015, 12/06/2019, 04/26/2020. Plots (**a**,**b**,**d**,**e**,**g**,**h**) are in linear scale and plots (**c**,**f**,**i**) in logarithmic scale (base 10). Supporting information S1 also includes: the vertical and the horizontal components of ground displacement at all analyzed GPS stations; the logarithmic plots of all seismic events and of their energy.

**Figure 4:**
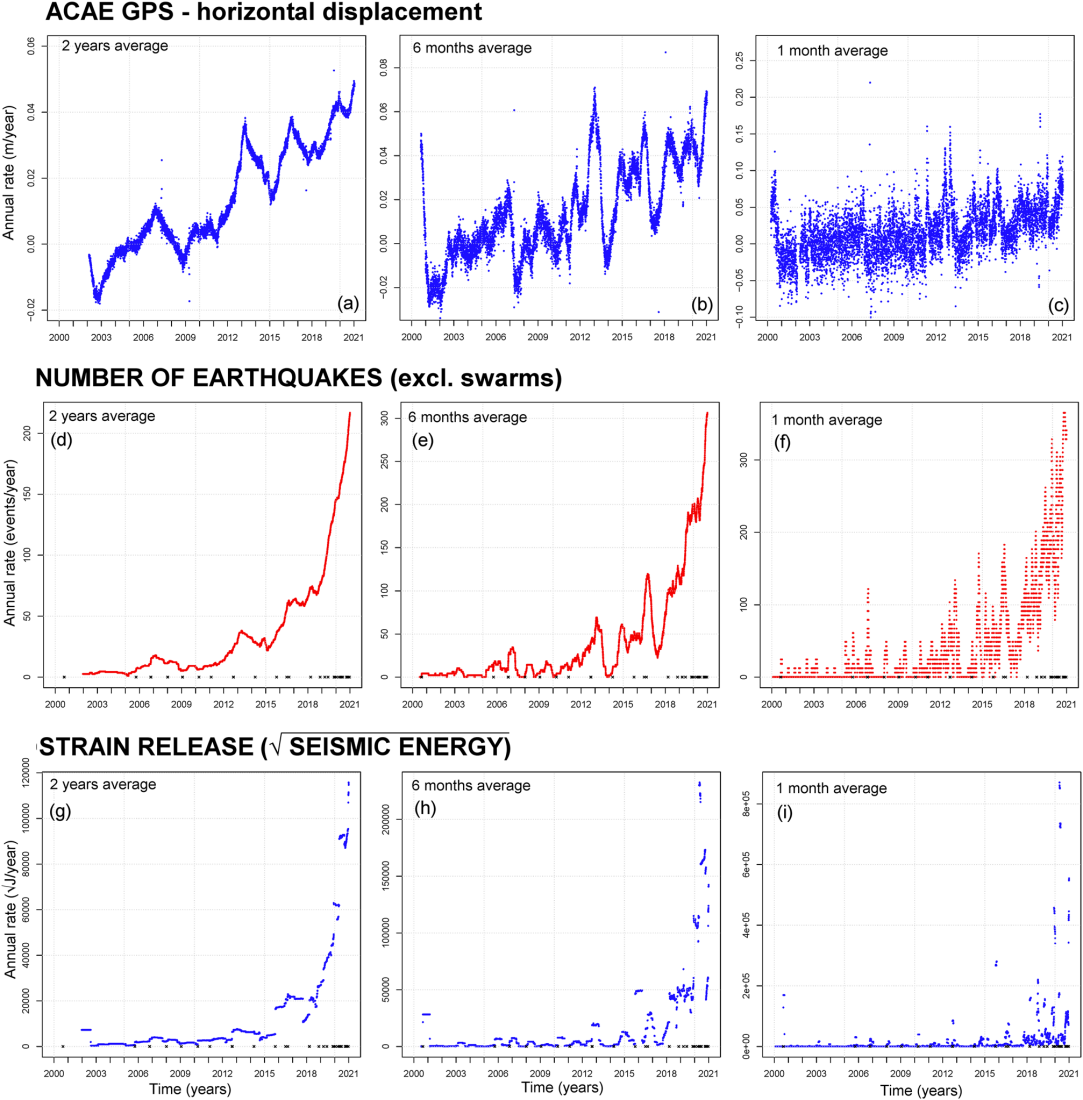
Examples of annual rate graphs. Plots (**a**-**c**) show the ground displacement rate; plots (**d**-**f**) show the annual rate of seismic events recorded (excl. swarms); plots (**g**-**i**) show the seismic strain rate estimation. Plots (**a**,**d**,**g**) are calculated on a 2-year moving average, plot (**b**,**e**,**g**) on a 6-month moving average, plot (**c**,**f**,**i**) on a 30-day moving average. In (**d**–**i**) the days with > 20 events are marked with black crosses along the x-axis. Supporting information S1 also includes: the annual rate of the vertical and horizontal components of ground displacement at all analyzed GPS stations; the annual rate of all seismic events and of their energy. These detail the 2-year, the 6-month, and the 30-day average results.

**Figure 5:**
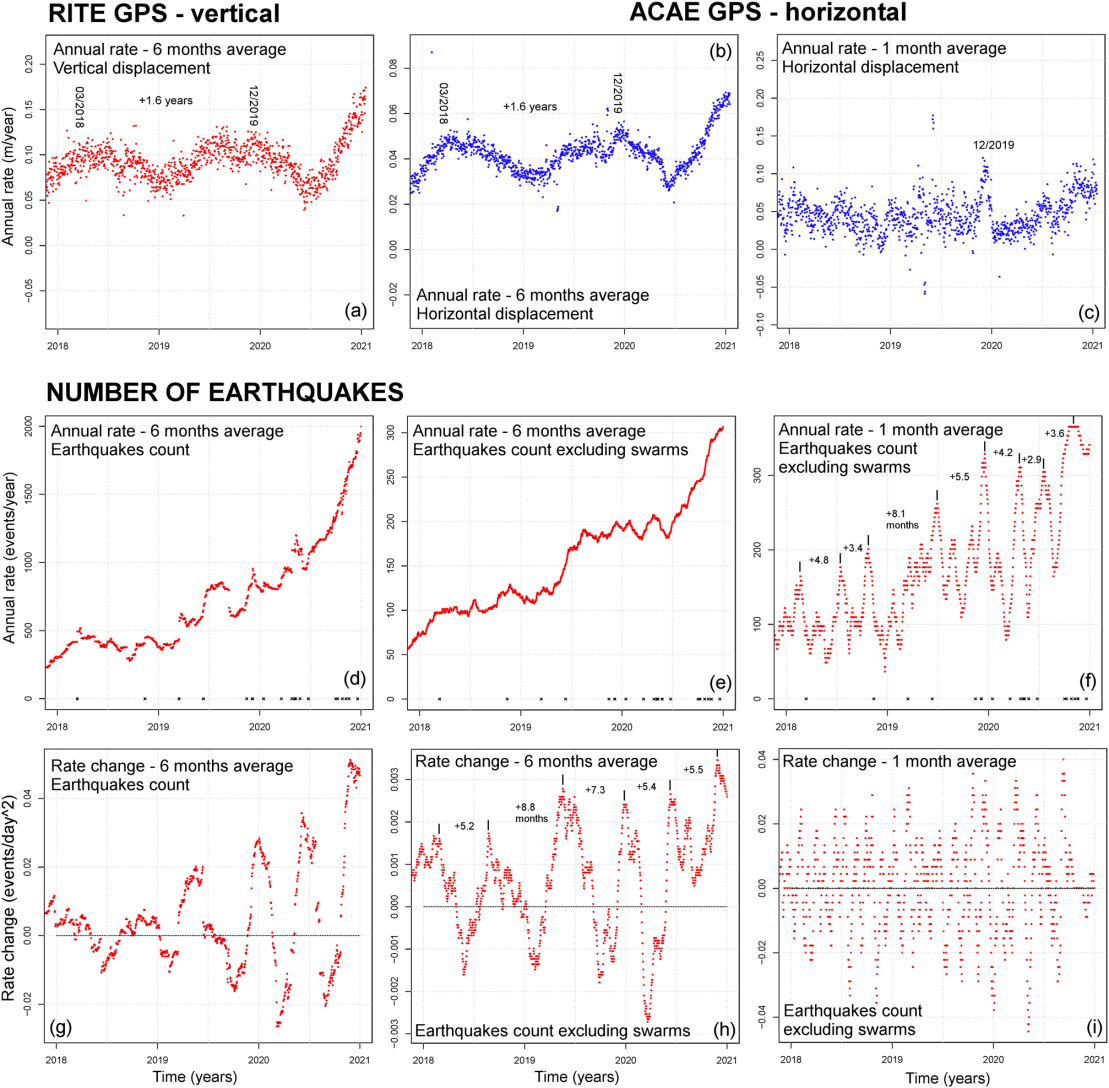
Details of the annual rate (**a**–**f**) and of the daily rate change (**g**–**i**) in 2018–2020. Plots (**a**,**b**,**d**,**e**,**g**,**h**) are calculated on 6-month moving average, plots (**c**,**f**,**i**) on 30-day moving averages. In (d-f) the days with > 20 events are marked with black crosses along the x-axis. Plots (**a**–**c**) show the ground displacement rate: plot (**a**) is the vertical displacement recorded at RITE GPS station; plots (**b**–**c**) are the horizontal displacement recorded at ACAE GPS station. Plots (**d**–**i**) show the seismic events recorded: plots (**d**,**g**) include all events in the catalog; plots (**e**,**f**,**h**,**i**) exclude the swarms, i.e. by counting no more than one seismic event per day. In (**a**,**b**,**f**,**h**) the main peaks are marked and the inter-peak times are labeled. Supporting information S1 also includes the annual rate and the daily rate change of the vertical and the horizontal components of ground displacement at all analyzed GPS stations, the seismic energy and the strain release. These detail the 2-year, the 6-month, and the 30-day average results.

**Figure 6:**
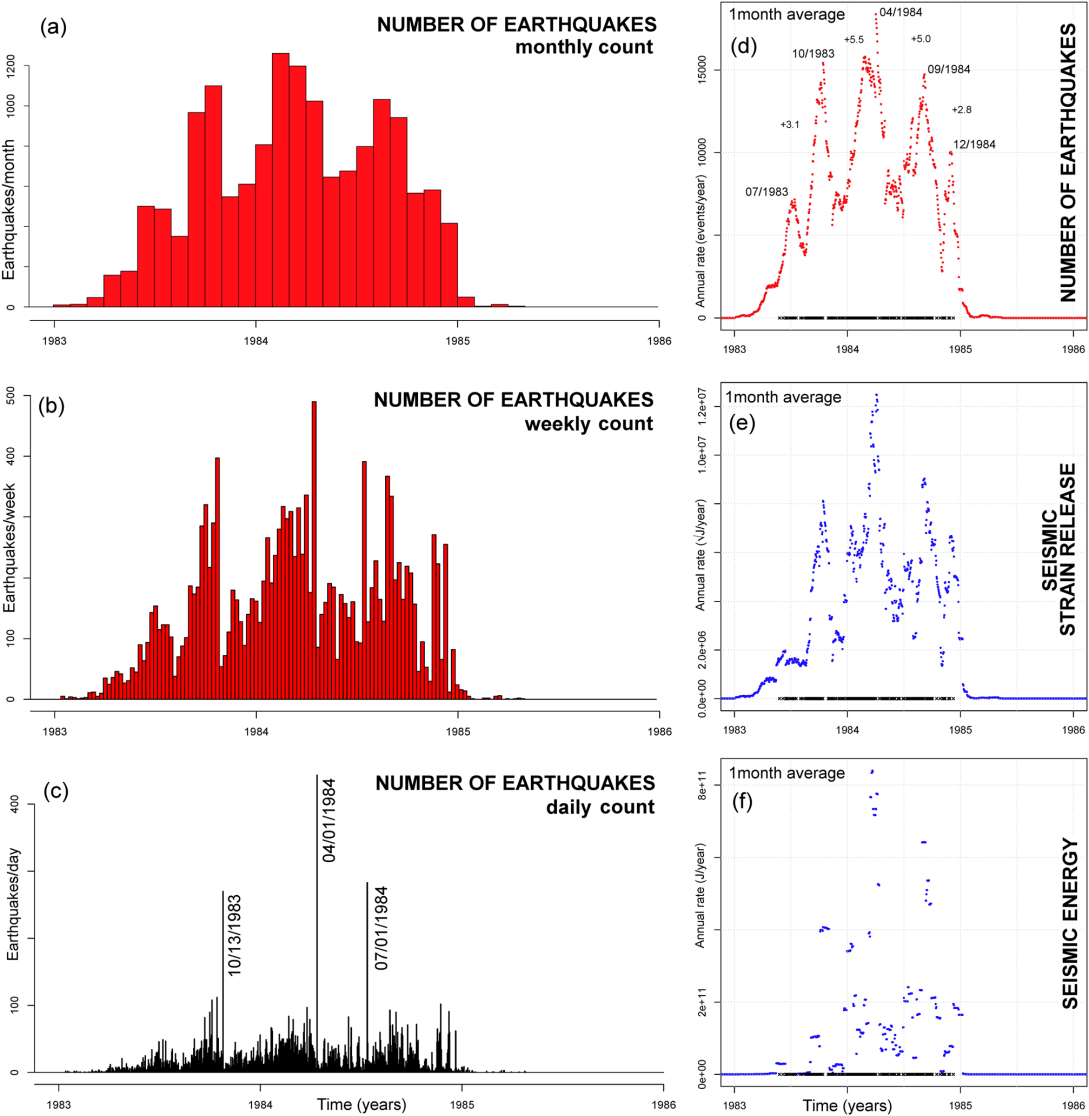
Overview of earthquakes recorded at Campi Flegrei in 1983–1985, including swarms. Plot (**a**) shows the monthly count, plot (**b**) the weekly count, plot (**c**) the daily count. The three greatest swarms, comprising more than 250 events, are labeled in (**c**). Plots (**d**–**f**) show the annual rate of (**d**) all seismic events recorded, (**e**) seismic strain release rate, and (**f**) seismic energy estimation. All rates are calculated on a 30-day moving average. In (**d**–**f**) the days with > 20 events are marked with black crosses along the x-axis. In (**d**) the main peaks are marked and the inter-peak times are labeled.

First, we describe the long-term trends in the data, from 2000 to 2020. In particular, after evaluating the data values (Fig. 3), we evaluate their annual rate (Fig. 4), i.e. displacement rate, seismic count rate, strain release rate. Then, we detail these results in the three years from 2018 to 2020 (Fig. 5), and for the seismic record from 1983 to 1985 (Fig. 6).

### Data

In Fig. 3 we introduce the time series that we investigate from 2000 to 2020. Figure 3a–c shows the ground displacement, with labels marking the mini-uplifts UP1-7^52^. Figure 3a shows the vertical displacement at RITE GPS, Fig. 3b–c to the horizontal displacement expressed in linear and logarithmic scale, respectively. There has been a vertical uplift of ~ 78 cm and a horizontal displacement of ~ 34 cm since 2005, of which ~ 90% has occurred since 2011, ~ 40% since 2018.

Figure 3d–f shows the earthquakes count, where Fig. 3d shows all events, with labels marking the size of the swarms of > 30 events. Figure 3e–f excludes the swarms and counts the events in linear and logarithmic scale, respectively. Removing the jumps produced by the swarms macroscopically affects the function shape in 2005–2014, but less afterwards. Besides, the total count decreases to ~ 900 events, of which ~ 90% have occurred since 2011, ~ 60% since 2018.

Figure 3g shows the cumulative seismic energy, and Fig. 3h–i the cumulative strain release in linear and logarithmic scale, respectively. Total seismic energy amounts to 3 GJ since 2000, of which > 97% has been released since 2015, ~ 80% since 2018. We label four significant events: 08/22/2000 (M_max_ 2.2)^25^, 10/07/2015 and 12/06/2019 (M_max_ 2.5 and 3.1)^68^, and 04/26/2020 (M_max_ 3.3), i.e. the strongest earthquake from 1985 to 2020. The strain release plot in Fig. 3h produces a function shape that is macroscopically similar to the earthquakes count, though several strain jumps are evident. Small jumps before 2015 are evident in Fig. 3i.

Although the three strands of data, i.e. horizontal displacement, seismic count without swarms, and seismic strain, are all accelerating, the logarithmic plots in Fig. 3c,f,i highlight significant differences between them. In particular, before 2015 in Fig. 3c the horizontal displacement is increasing, but erratically for the mini-uplifts. Since 2015, both the horizontal displacement and seismic count have almost been linear in log-scale, but the former is still concave-shaped, the latter, shown in Fig. 3f, is convex-shaped, i.e. growing faster than an exponential function. In Fig. 3i the seismic strain is always convex-shaped and significantly accelerating also in log-scale, possibly with a hyperbolic trend.

### Annual rate s

In Fig. 4 we display the annual rate of the data from 2000 to 2020. We base the estimate of the annual rate on left-side first-order finite differences. We use left-side intervals so that the value at time t is not anticipating future information^16,19,23^: F'(t) = [F(t) −  F(t-h)] / h.

Since the data are significantly noisy on short time scales, the finite difference approximation remarkably changes depending on the time step selected. We produced rates at 2-year (Fig. 4a,d,g), 6-month (Fig. 4b,e,h), and 30-day (Fig. 4c,f,i) time steps, corresponding to the average rates on moving windows of these lengths. We note that the annual rate can track the periods of longer and more intense ground movement and seismicity. These derived data emphasize the oscillations that characterize the analyzed time series.

Figure 4a–c displays the horizontal displacement at ACAE GPS station. A linear least squares fit from 2000 to 2020 produces a linearized rate slope, i.e. acceleration, of 0.3 cm/yr^2^ regardless of the time step selected. Almost the same result is obtained from 2011 to 2020. In contrast, from 2000 to 2010, the least square fit produces a lower linearized rate slope of 0.15 cm/yr^2^ in Fig. 4a,b, and 0.05 cm/yr^2^ in Fig. 4c. The linearized fit of the vertical displacement rate at RITE GPS station is 0.6 cm/yr^2^ and shows similar features to the rate of the horizontal displacement at ACAE GPS. The graphs possess significantly more noisy features before 2011, and show an alternation of evident speed-ups and slow-downs at various frequencies. These speed-ups include the mini-uplifts UP1-7 and then several other oscillations that have occurred since 2015.

Figure 4d-f presents the seismic count excluding swarms. In Fig. 4d the maximum rate is at about 210 events/year, in Fig. 4e it reaches 300 events/year, in Fig. 4f. 350 events/year, all observed at the end of 2020. There have been more days with > 20 events since 2018, i.e. 20, than from 2000 to 2017, i.e. 14. These graphs show an alternation of speed-ups and slow-downs on the short term, such as the ground displacement, but in this case, the overall rate trend is accelerating nonlinearly.

Figure 4g-i shows seismic strain release. In Fig. 4g the annual rate surpassed 10,000 √J/year on a 2-year average, and the plot is macroscopically similar to the seismic count rate. However, concentrated peaks corresponding to the strongest earthquakes intermittently contribute to the total strain release, as in Fig. 4h,i.

In addition, Supporting Information S2 shows the rate change of the data, based on the left-side second order finite differences^14^: F"(t) = 4[F(t) + F(t-h) − 2F(t-h/2)] / h^2^.

Similar to the annual rate plots, we produced rate changes with 2-year, 6-month, and 30-day time steps.

All rate change plots highlight the alternation of positive and negative rate changes. Since the GPS rate changes lack of a significant trend, we build the Fourier analysis on these functions, as described in “Fourier spectrum of the GPS data in 2000–2020”. Seismic count and seismic strain also show an alternation of positive and negative rate changes, like for ground displacement, but the amplitude of these oscillations increases with time, and their positive part still shows an average increasing trend which hinders Fourier analysis.

### Details of the data collected in 2018–2020

In Fig. 5 we display the annual rate and the rate change of the data from 2018 to 2020. Because of the short timeframe we only produced average rates with 6-month (Fig. 5a,b,d,e,g,h), and 30-day (Fig. 5c,f,i) time steps.

Figure 5a–c shows ground displacement data. In Fig. 5a,b the graph displays an alternation of two speed-ups and slow-downs, with a period of about 1.6 years between the two maxima at 03/2019 and 12/2019. In Fig. 5c the graph shows a significant peak in 12/2019, followed by a decrease and a new increase during year 2020.

**Figure 7:**
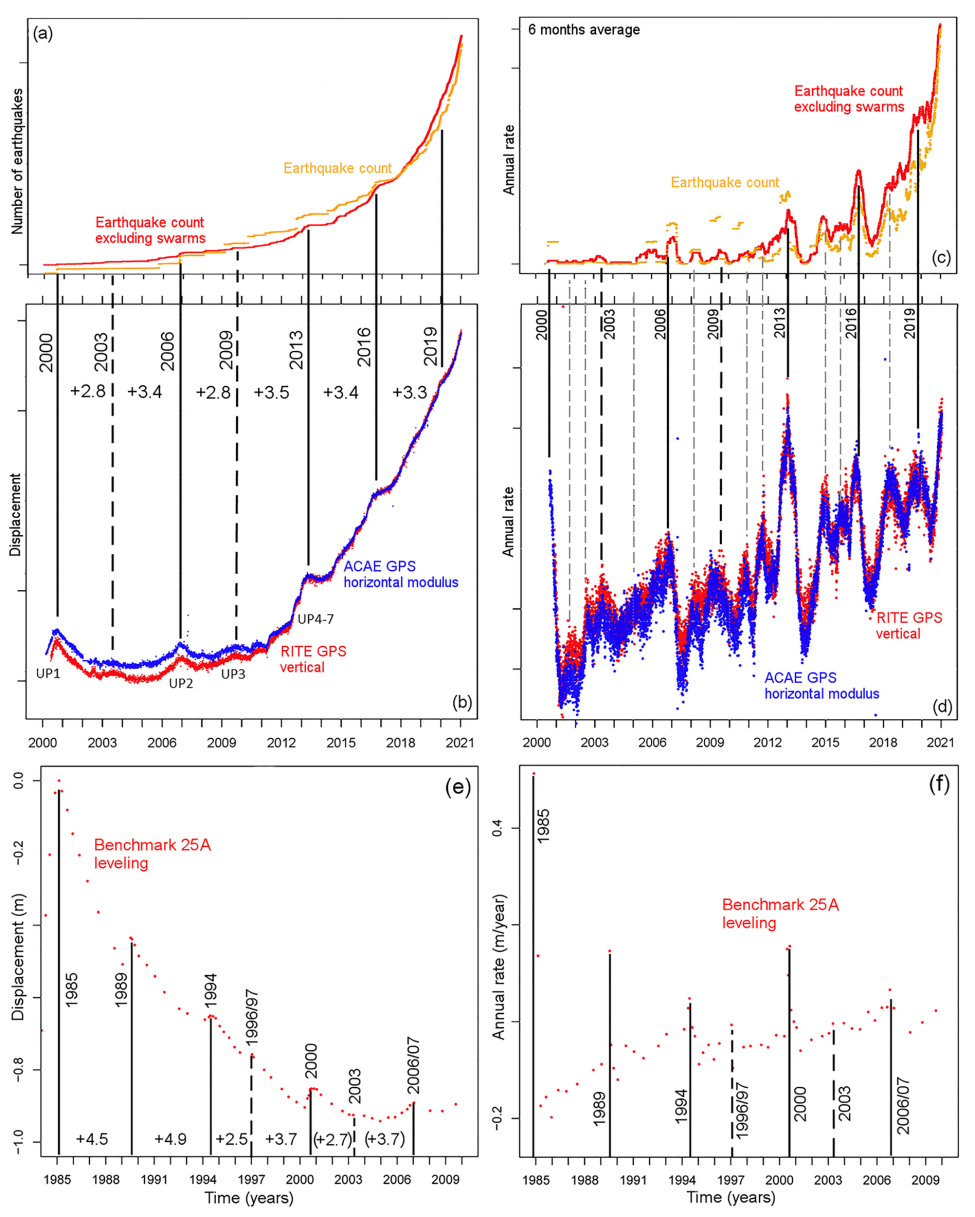
Plot (**a**–**d**) form a synoptic panel comparing (**a**,**c**) seismic count and (**b**,**d**) ground displacement in 2000–2020. In plots (**a**,**c**) the results including all events in the catalog are orange, and those excluding swarms are red. In plots (**b**,**d**) the vertical displacement recorded at RITE GPS station is red, and the horizontal displacement recorded at ACAE GPS station is blue. Plot (**e**–**f**) shows the leveling data recorded at benchmark 25A in 1985–2010. Plots (**a**,**b**,**e**) show monitoring data and plots (**c**,**d**,**f**) show their annual rate. In all plots the main peaks are marked with black vertical lines and labeled with the corresponding year. In plots (**b**,**e**) the inter-peak times are labeled. Dashed black lines mark the least evident main peaks. In plot (**d**) dashed grey lines mark some of the secondary peaks.

**Figure 8:**
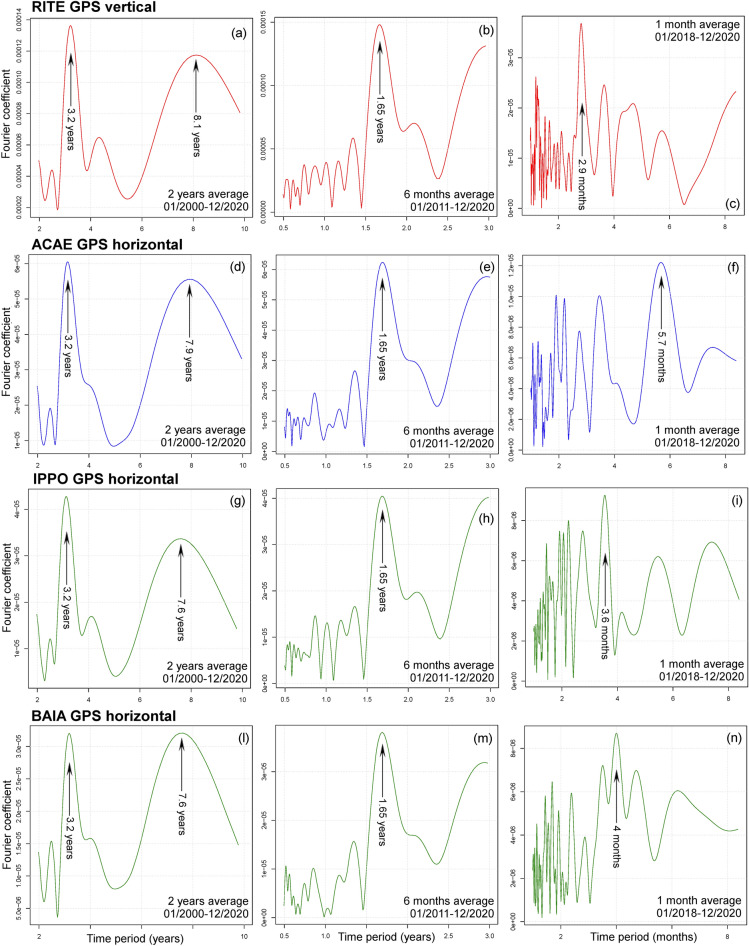
Examples of Fourier spectrum of the ground displacement GPS data. Plots (**a**–**c**) show the vertical displacement recorded at RITE GPS station; plots (**d**–**n**) show the horizontal displacement recorded at (d-f) ACAE, (**g**–**i**) IPPO, and (**l**–**n**) BAIA GPS stations. Plots (**a**,**d**,**g**,**l**) are calculated on a 2-year moving average in 2000–2020, plot (**b**,**e**,**g**,**m**) on a 6-month moving average in 2011–2020, plot (**c**,**f**,**i**) on a 30-day moving average in 2018–2020. Main time periods are marked and labeled. Supporting information S1 also includes the Fourier spectrum of the vertical and the horizontal components of ground displacement at all analyzed GPS stations. These detail the 2-year, 6-month, and 30-day average results obtained in 2000–2020, 2011–2020, 2018–2020.

Figure 5d–i shows the seismic count, with Fig. 5e,f,h,i excluding the swarms. In Fig. 5d,e the long term trend partially hides the periodic features. However, despite the acceleration, Fig. 5e shows two speed-ups and slow-downs consistent with the GPS data. In Fig. 5f–h, an alternation of minima and maxima is very evident, with variable periods of 3 to 8 months in Fig. 5f and 5–8 months in Fig. 5g,h. We note that the maxima of all these oscillations reach higher values with time. Figure 5i captures very fast and mixed harmonics, difficult to distinguish.

### Overview of the seismic record collected in 1983–1985

Figure 6A shows the monthly number of earthquakes recorded during the most-recent bradyseismic crisis of 1983–1984 and the very few events occurred in 1985. Similar to the previous subsection, in Fig. 6b–f we focus on a three-year interval, 1983–1985. Figure 6c shows that the bradyseismic crisis included three significant swarms in 10/13/1983, 04/01/1984, 07/01/1984, with the first and third swarm including > 250 events, and the second > 400 events. Note that the distribution of the epicenters of the 1983–1984 crisis was very different from that observed after 2000, as described in De Siena et al.^58^ and reported in “Physical interpretation of CFc unrest” section.

Figure 6d–f shows the annual rate of the number of earthquakes (Fig. 6d), their strain release (Fig. 6e) and seismic energy (Fig. 6f), averaged at 30-day time steps. Figure 6d–e display five peaks, at 07/1983, 10/1983, 04/1984, 09/1984, 12/1984. These peaks resemble the speed-ups and the following slow-downs observed in the recent data (Fig. 5f), although the rates are in this case an order of magnitude larger. The intervals between the peaks are ~ 3 months for the first and the fourth, and ~ 5 months between the middle peaks, similar to what seen in Fig. 5f–h. Figure 6f displays the three peaks observed in Fig. 6c, confirming that energy release is well coupled with event counts. In fact, the most energetic events were always associated with a large number of smaller events.

## Recurrent oscillations in 1985–2020

Following the analysis of rates, we next focus our attention on the features of the speed-ups and slow-downs that characterize both GPS and seismic data. In particular, we show a synoptic panel that displays the consistency of the alternation of minima and maxima in the two types of data in 2000–2020. We observe similar harmonics in the leveling data collected in 1985–2010 (Fig. 7). Then, we present an estimation of the Fourier spectrum of the annual rate of ground displacement data (Fig. 8). We note that these oscillations mark periods of increased stress and damage of the ground, which could be amplified in the future in case of a continuation of the decennial-like accelerating inflation trend (see below for more discussion).

### Synoptic panel of ground deformation and seismic data in 1985–2020

In Fig. 7 we show a synoptic panel describing the alternation of minima and maxima in GPS and seismic data. Figure 7a,b compares synchronous records of seismic count, with and without swarms, to the vertical component of RITE GPS and the horizontal component of ACAE GPS in 2000–2020. We marked the maxima in 2000, 2003, 2006, 2009, 2013, 2016, 2019, their inter-maxima being 2.8, 3.4, 2.8, 3.5, 3.4, 3.3 years, i.e. 3.2 years on average. Among these, 2003 and 2009 are less evident than the others and if were excluded, they would have produced two inter-maxima of 6.2 and 6.3 years^113^. Figure 7c,d shows the annual rates on a 6-month average, which highlight the seven main peaks described in Fig. 7a,b. We also observe number of secondary peaks between each of the previous. In particular, they split 2000–2003 and 2009–2013 in three similar sub-intervals, 2003–2006, 2006–2009, 2016–2019 in two similar sub-intervals, and 2012–2016 in three sub-intervals where the first is about twice the second and third. These integer proportions highlight the complex harmonics that sum up in the signal.

Figure 7e shows leveling data collected at benchmark 25A in 1985–2010^51^. We marked the maxima in 1985, 1989, 1994, 1996/97, 2000, 2003, 2006/2007, where Fig. 7a–d more accurately describes the last three. Among these, 1996/97 and 2003 are less evident than the others. The times between the first five maxima are 4.5, 4.9, 2.5, 3.7 years; i.e. the two first intervals were 40–50% longer than average. Figure 7f shows the annual rate based on linear increments between consecutive leveling values. These confirm the maxima described in Fig. 7e. Secondary more frequent maxima are not possible to define from these discrete measurements.

### Fourier spectrum of the GPS data in 2000–2020

Figure 8 displays the Fourier Transform of the annual rate of the GPS signals collected at four different stations—RITE, ACAE, IPPO and BAIA (Fig. 1a). Since the annual rate F' displays an increasing trend (Fig. 4a,b) we consider the rate change F"(t) instead, and by ∀k in ℕ we define the Fourier coefficients:

$$ \Phi \left( {k, \, F^{\prime } } \right) = [\varphi_{1} \left( k \right)^{2} + \varphi_{2} \left( k \right)^{2} ]^{1/2} / \, k, \varphi_{1} \left( k \right) = \sin_{k} \cdot F^{\prime \prime } , \varphi_{2} \left( k \right) = \cos_{k} F^{\prime \prime } , $$

where $\cdot$ is the integral product over the interval [t_0_, t_0_ + Δt], and ∀t in the interval we have:

$$ {\text{sin}}_{{\text{k}}} \left( {\text{t}} \right) \, = {\text{ sin}}\left( {{2}\pi {\text{kt }}/ \, \Delta {\text{t}}} \right), {\text{cos}}_{{\text{k}}} \left( {\text{t}} \right) \, = {\text{ cos}}\left( {{2}\pi {\text{kt }}/ \, \Delta {\text{t}}} \right). $$

That is, first we calculate the Fourier Transform of F" and then we derive the Fourier Transform of F' by:

$$ \Phi \left( {{\text{k}},{\text{ F}}^{\prime } } \right) \, = \Phi \left( {{\text{k}},{\text{ F}}^{\prime \prime } } \right) \, /{\text{ k}} $$

In “Recurrent oscillations in 1985–2020” section we calculated the annual rate F' and the rate change F" according to three time steps, i.e. 2-year, 6-month, 30-day, which focus on different parts of the Fourier spectrum. In fact, the finite differences kill any harmonics with a period equal to the time step or its integer dividers, thus altering the spectrum below it. Since the noise affecting F'' decreases with time, we set different domains [t_0_, t_0_ + Δt] with the three adopted time steps.

Therefore, Fig. 8a,d,g,l uses a 2-year time step to show Fourier coefficients from 2 to 10 years in 2000–2020; Fig. 8b,e,h,m uses a 6-month time step to show coefficients from 0.5 to 3 years in 2011–2020; Fig. 8c,f,I,n uses the 30-day time step to show the coefficients from 1 to 8 months in 2018–2020. Supporting information S1 includes additional plots that detail other combinations of time domain and part of the Fourier spectrum, thus testing the sensitivity of the main harmonics on the time domain selected.

In particular, Fig. 8a,d,g,l shows two main peak Fourier coefficients at 3.2 years and 7.6–8.1 years. The first value is the average period observed in Fig. 7a–d. The second value is about 5/2 times greater, and it is associated with a wide interval of high values in the Fourier spectrum, from 6.5 to 9 year-periods, enveloping both twice and three times the 3.2 year peak. We note that, in our application, Fourier analysis may not accurately capture longest periods, because it partially interprets the nonlinear accelerating trend as the beginning of a long wave. However, excluding the least evident peaks in Fig. 6 can motivate such a long period.

Figure 8b,e,h,m displays a peak at 1.65 years, which is about half the main period at 3.2 years, also apparent in this plot. The shorter period is consistent with some of secondary maxima observed in Fig. 7c,d. Finally, Fig. 8c,f,l,n describes a less regular recurrence for the shortest harmonics—peaks at 2.9, 3.6, 4, and 5.7 months are apparent in the data of different GPS stations. The main peaks measured in one spatial location are also clearly discernible as a secondary peak in some of the other sites, suggesting a superimposition of multiple spatially irregular frequencies.

## The FFM tool and its probabilistic formulation

The Failure Forecast Method (FFM) for volcanic eruptions is a classical tool applied in the interpretation of geophysical monitoring data as potential precursors, providing quantitative predictions of the “failure time”, i.e. the time when the system could reach a critical state conditional to the continuation of the same accelerating trend^116,117,119^. The method is based on a nonlinear regression of the temporal rate X of the signals, according to the equation:

$$ {\text{dX}}/{\text{dt }} = {\text{ A X}}^{\alpha } , $$

where A > 0, and α is in [1.2, 2.0] in our case study^71–73^. This approach is valid under the assumption that the previously observed nonlinear trend of X will continue in the future, and accelerate in the same way as elastic-brittle materials subject to a constant stress do while approaching their rupture. Therefore, seismicity and ground deformation are the type of signals most extensively studied with the FFM method in volcanology, producing often good results in the retrospective analysis of monitoring signals preceding explosive eruptions of various size^31,36,46,48,104,118,120^. For instance, at basaltic calderas, ground deformation rate proved to be a good indicator of the probability of magma transfer towards the surface, and thus eruption, week or months in advance^63^. Deviation from the elastic-brittle assumption could cause decoupling between seismic and deformation trends^75^, as discussed in “Results application and interpretation” section. In this study, since most of the seismic events have similar low magnitude (see Fig. 2), the few most energetic events dominate the amount of strain release. In contrast, seismic count treats all events as equal, and it was already adopted in several cases to fully describe the unrest evolution in CFc^41,42^. For this reason, we analyzed with FFM both seismic count and strain release datasets, and described the difference in the results section.

In more detail, in the original equation the change of variables η = X^1−α^ implies:

$$ d\eta /dt \, = \, \left( {1 - \alpha } \right)A, $$

i.e., the solution η is a line, which hits zero at time t_f_, i.e. the failure time. If α = 2 the inverse rate is linear. The most commonly used graphical and computational methods rely on analysis of the inverse rate 1/X^46,47^.

The main sources of uncertainty in the FFM are: (i) the value of the exponent α that defines the nonlinear curvature of the observed data; (ii) the confidence interval related to the regression itself^6,7,29,115^. Therefore, Bevilacqua et al.^17^ enhanced the method, and utilized the residuals of the regression to define a stochastic noise and incorporate it in the nonlinear equation using a mean-reversion mechanism. This improved the statistical accuracy and enabled the calculation of a more robust probability density function for the failure time. However, as better discussed in the following sections, this method is not immune to false or missed alarms, and future variations in monitoring data could always change the observed trends (see for instance Bevilacqua et al.^17^, for more details).

Note that reaching a “critical state” does not necessarily mean the occurrence of an eruption, the “failure time” could also represent a tipping point for the system, changing its physical structure. For example, many sequences of short-term accelerating seismic signals were recorded during caldera collapse following the drainage of the lava lake at Pu’u ‘O’o, Kilauea, Hawai’I, in 2018^85^, corresponding to an alternation of stress build-up and material failure, not associated with large scale explosions^106^. In the case of CFc, a tipping point might be representative of the decrepitation of a superficial magma system; from this moment onwards the system could evolve with a new process that could in turn have its own acceleration trend likely described by a new and different FFM^96^. Nevertheless, the FFM provides an estimate for the upper limit for the possible future continuation of the observed nonlinear acceleration until the system does not change that trend, either gently or abruptly.

We remind that the FFM analysis described in this section is complementary and independent from the previous analysis of recurrent oscillations. In fact, the two approches describe two overlying trends with different time scales—decennial versus annual/month-like. However, the FFM approach based on a 3-year regression may read the increasing phase of oscillations with period above 6 years as an accelerating trend.

### Example of FFM application on 1 Jan 2021

Through the analysis of the inverse rates we first focus on an example of application of FFM to the GPS and seismic data at 1 Jan 2021. In particular, we follow the probabilistic formulation of Bevilacqua et al. ^17^, i.e. a nonlinear regression and stochastic extrapolation. In Supporting Information S3 we display the inverse rate of the data, i.e. 1/F'. Similar to the annual rate plots, the finite difference approximation highlights various multiscale properties depending on the time step selected. We produced inverse rates using time steps at 2-year, 6-month, and 30-day time steps. We note that the transient phases of acceleration produce local minima in the plots.

**Figure 9:**
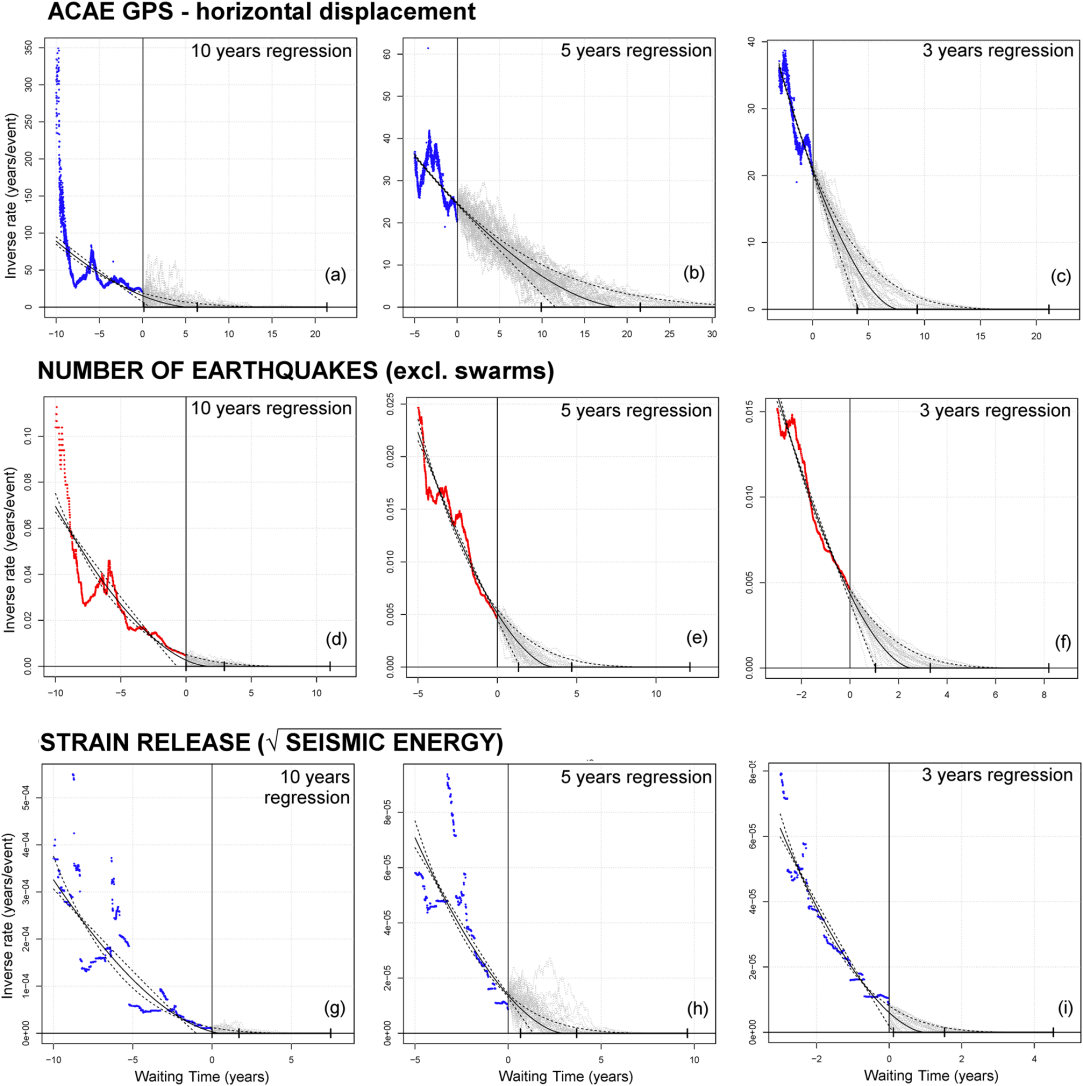
Examples of pFFM regression plots and stochastic paths. The forecast is formulated at t = 01/01/2021, marked by a vertical line. The failure time t_f_ is expressed in terms of the waiting time (t_f_ − t). On the left, the colored dots show the inverse rate data. On the right, gray dotted lines show 50 random stochastic solutions. A solid black curve shows the mean FFM regression and dashed lines mark its 90% uncertainty range. A bold interval on the x-axis marks the mean and the 90% confidence of the pFFM forecast. Plots (**a**,**d**,**g**) use a 10-year regression, plots (**b**,**e**,**h**) a 5-year regression, plots (**c**,**f**,**i**) a 3-year regression. Plots (**a**–**c**) are based on the inverse rate of ACAE GPS ground displacement; plots (**d**–**f**) on the seismic events recorded (excl. swarms); plots (**g**–**i**) on the seismic strain release estimation. All inverse rates are calculated on a 2-year moving average. Supporting information S1 also includes: the pFFM examples based on the vertical and the horizontal components of ground displacement at all analyzed GPS stations; all seismic events and of their energy. These detail the 2-year, 6-month, and 30-day average rate results, and the 10-year, 5-year and 3-year regression.

Figure 9 displays the nonlinear regression of the inverse rate, expressed with three lines marking the mean value and the 5th and 95th percentiles of the confidence interval of slope A and intercept x_0_, and the uncertainty range of the curvature α, that we uniformly sampled in [1.2, 2.0]. This choice of the range of α is consistent with linear or sublinear decrease of the inverse rate^48^. The future extrapolation is performed through a stochastic path driven by the regression line, but incorporating perturbations that fit the residuals observed in the past (mean reversion parameter ϒ = 1/2 year)^17^.

We also tested the sensitivity of the results on the length of time regression in the past, to explore the accelerating trend at various time scales. Figure 9a,d,g uses a 10-year regression, Fig. 9b,e,h uses a 5-year regression, and Fig. 9c,f,i-a 3-year regression. Other regressions could be tested, e.g. 1-year or 15-year, which may capture short-term variations or fit the entire uplift since 2005, but we do not expect them to differ significantly from the 3-year and the 10-year cases, respectively. All the examples are based on the 2-year averaged annual rates, but Supporting information S1 includes the 6-month and 30-day average rate results. These additional plots are overall consistent with Fig. 9, but more sensitive to quick changes in the signals and prone to stronger influence of noise.

In Fig. 9a–c, we have failure times estimates in [0, 6, 21] years, [10, 21, > 25] years, [4, 9, 21] years from 01/01/2021, respectively based on 10-year, 5-year, and 3-year regressions. In Fig. 9d–f, we have [0, 3, 11] years, [1, 5, 12] years, [1, 3, 8] years, with forecasts significantly more steady than in the ground displacement case. In Fig. 9g–i, we have [0, 1, 7] years, [0, 4, 10] years, [0, 1, 5] years, respectively—the shortest that we obtained.

In addition, Supporting Information S4 displays the estimates of the failure time expressed in terms of the monthly PDF of the waiting time from 1 Jan 2021. We calculated the 5^th^ percentile, mean value, and 95^th^ percentile of the waiting times and of the monthly PDF values, in all the various examples. We note that some PDFs show peaks at [2, 10]% in the first months, but then they decrease to lower values, i.e. [0.5, 2.0]% in the most of the remaining interval.

### Sensitivity analysis of the FFM forecasts

**Figure 10:**
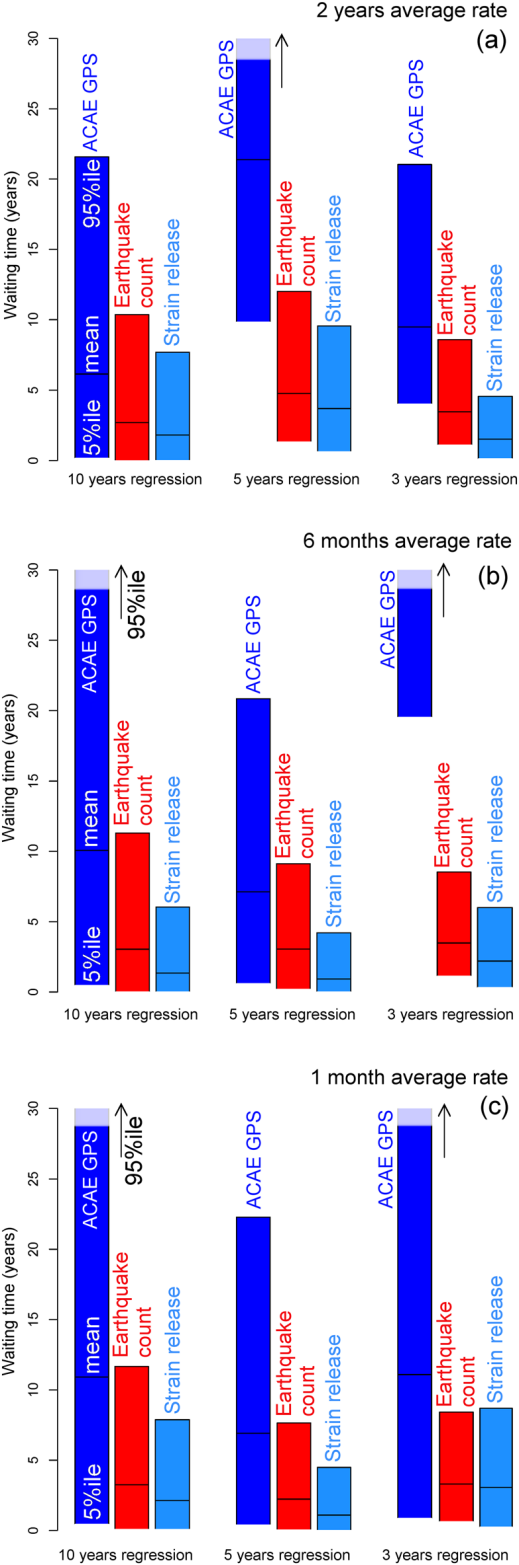
Barplot of the waiting time obtained with the pFFM applied to the horizontal ACAE GPS displacement, seismic count excluding swarms and strain release data. The forecast is formulated at t = 01/01/2021. The bars show the mean and the 90% confidence of the pFFM forecast. Different data are marked with different colors and labeled accordingly. Examples are based on 10-year (left), 5-year (center), and 3-year (right) time regression. Plot (**a**) uses a 2-year moving average, plot (**b**) a 6-month moving average, plot (**c**) a 30-day moving average.

Figure 10 shows the sensitivity analysis of the failure time t_f_ characterizing the different seismic data and time step in signals’ rate calculation. In particular, we show the waiting times (t_f_ − t), where t is equals to 01/01/2021 for all the data analyzed, i.e. horizontal ACAE GPS displacement, seismic count excluding swarms, strain release. Figure 10a uses a 2-year time step, Fig. 10b uses a 6-month time step and Fig. 10c uses a 30-day time step, to define the signals’ rate.

The horizontal displacement produces significantly longer waiting times than the seismic data, i.e. an observation that may indicate the transition from an elastic-brittle to a quasi-elastic regime of deformation, as discussed in “Results application and interpretation” section. In Fig. 10a the 5-year regression, and in Fig. 3b the 3-years regression, of ground displacement highlight longer waiting times than the other regressions. For the seismic data, all the regressions are consistent. Strain release produces similar or shorter waiting times than the seismic count, because the most energetic events, i.e. with magnitude > 3.0, only appeared in 2019–2020. Supporting Information S5 includes additional barplots for the total seismic count (including swarms) and the energy release.

Finally, Supporting Information S6 shows the spatial analysis of probabilities P_2_, P_5_, P_10_, P_25_ of waiting times lower than 2, 5, 10, and 25 years, respectively, in the eleven GPS stations analyzed^20,21^. We preferred to calculate these probabilities, rather than the uncertainty range of waiting times, because its upper bound is greater than 30 years in almost every case. We classified the stations in three groups, according to their distance from the center of deformation. They are 1 “central” station (RITE), 4 proximal stations (< 3 km from RITE), and 6 distal stations (Fig. 1a)^18,54^. We note that the RITE station and all the 4 proximal stations produce similar estimates to those of ACAE shown in Fig. 10. Based on a 10-year regression, failure time probability is 40–60% in 5 years, and 65–75% in 10 years, on average. Instead, the 6 distal stations produce smaller or more uncertain estimates, especially for vertical displacement data, because in their signals the inflation is at the same scale of ambient noise^22,23^.

### Temporal evolution of forecasts in 2000–2020

**Figure 11:**
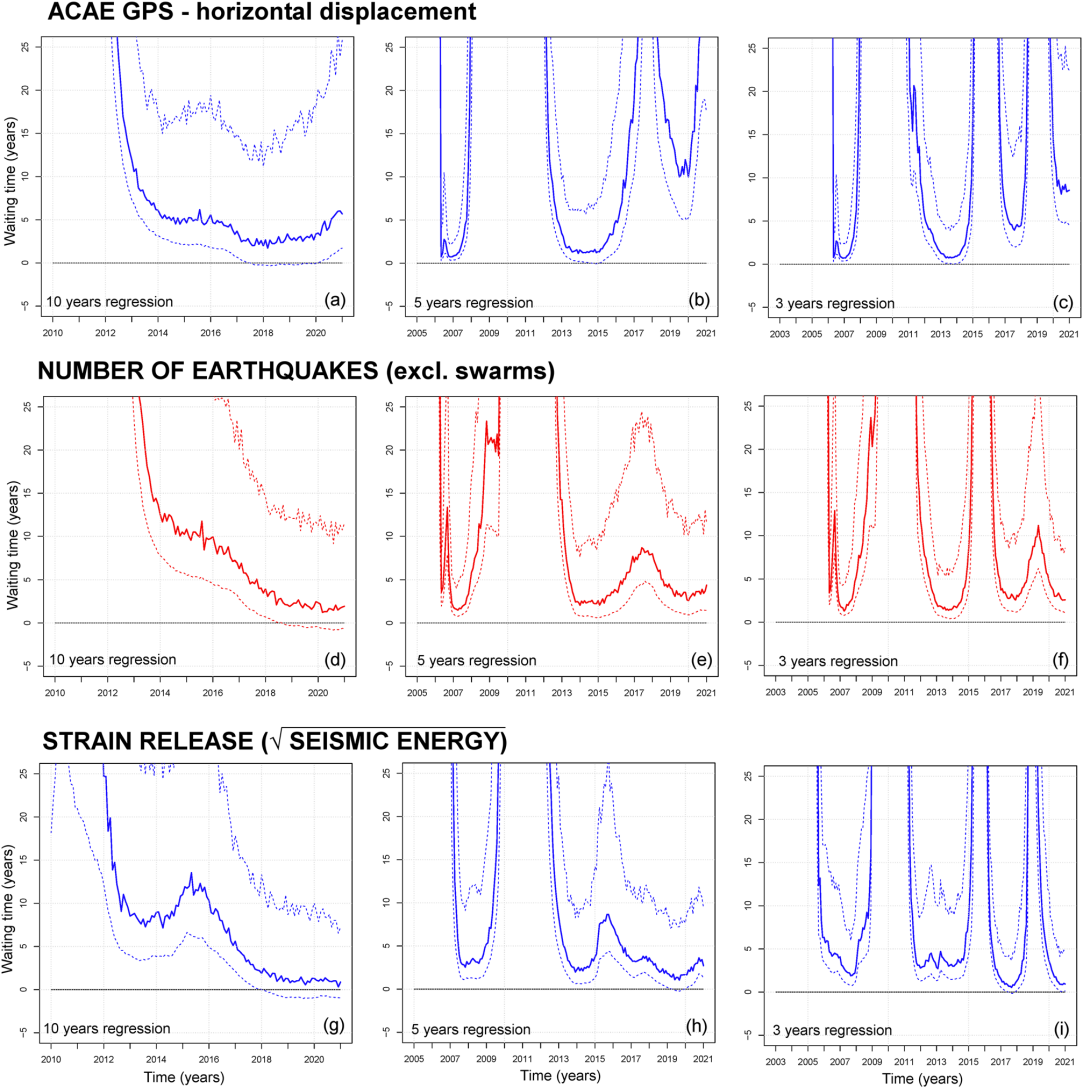
Retrospective analysis of the waiting time [t_f_(t) − t] from 2000 to 2020, as a function of the time t at which the forecast was formulated. The bold colored line is the mean forecast and the dashed lines are the 5th and 95th percentiles of its uncertainty range. Plots (**a**,**d**,**g**) use a 10-year regression, plots (**b**,**e**,**h**) a 5-year regression, plots (**c**,**f**,**i**) a 3-year regression. Plots (**a**–**c**) are based on the inverse rate of ACAE GPS ground displacement; plots (**d**–**f**) on seismic events recorded (excluding swarms); plots (**g**–**i**) on seismic strain estimation. All inverse rates are calculated on a 2-year moving average. Supporting information S1 also includes: FFM examples based on vertical and horizontal components of ground displacement at all analyzed GPS stations; all seismic events, and their energy. These plots detail the 2-year, 6-month, and 30-day average rate results, and the 10-year, 5-year and 3-year regressions. In addition, S1 includes all the results expressed in terms of the failure time t_f_.

Figure 11 summarizes how the FFM forecast t_f_(t) evolved from 2000 to 2020 as a function of the time t at which it was formulated. Similar to Fig. 10, we express the failure time t_f_(t) in terms of the waiting time [t_f_(t) − t] and set our domain to values < 25 years. Because the waiting time is the difference between the current time and the failure time, it appropriately describes the evolution of the FFM forecast. In particular, ∀t we show the 5^th^ percentile, the mean value, and the 95th percentile of [t_f_(t) − t]. All inverse rates are calculated on a 2-year moving average, and Supporting Information S7 and S8 show the additional results based on 6-month and 30-day moving average. These plots are more sensitive to quick changes in the signals and to the noise, but overall consistent (see Fig. 9 for a detailed comparison on 01/01/2021).

Figure 11a–c shows the ACAE GPS horizontal displacement, Fig. 11d–f shows seismic counts excluding swarms, and Fig. 11g–i shows the seismic strain release. The time domain starts from 2000 + R, where R is the implemented regression, hence it differs between the figures. We note that an ideal system perfectly consistent with the FFM equations would produce a straight line in Fig. 11, with slope -1. For simplicity, in this retrospective analysis we did not implement stochastic noise effects.

Figure 11a,d,g uses a 10-year regression. The waiting times decrease in mean value after 2012, except for Fig. 11a during 2020, and Fig. 11g in 2014–2017. In 2012–2017 the mean values in Fig. 11a are smaller than in 11d,g, while after 2018 it is the opposite. These trends illustrate the decennial long-term acceleration that characterizes the main dynamics from 2012 to 2020.

Figure 11b,e,h uses a 5-year regression. We show two phases of reduced waiting times—in 2006–2009 and then in 2012–2020, the latter already detailed in Fig. 11a,d,g. In Fig. 11b the first phase ends earlier, in 2008. Moreover, the second phase splits in two parts, with a gap in 2017. This last part, in 2018–2020 is characterized by waiting times that, in mean value, are never below 10 years, and display a sharp increase in 2020. Figure 11e,h are similar, and both show a slight increase in waiting time in 2020. However, Fig. 11e shows a temporary increase in the waiting time in 2015–2017, while in Fig. 11h it only lasted until the end of 2015.

Figure 11c,f,i uses a 3-year regression. We show three-four phases of short waiting times—2006–2007, 2011–2013, 2016–2020. In Fig. 11c,i the third phase clearly splits in two parts, 2016–2017 and 2019–2020. Figure 10c is characterized by an earlier conclusion of the first phase, in 2008, as observed in Fig. 11b. Moreover, the two parts of the third phase, in mean value, are never below ~ 5 years and ~ 9 years, respectively. Their gap is longer than in Fig. 11i. Figure 11f,i are very similar. However, in Fig. 11f the mean values of minimum waiting times in the second phase are lower than afterwards, while in Fig. 11i the opposite occurs.

## Results application and interpretation

In the following sub-sections, we discuss some physical interpretations of the observed features based on existing theories as well as the strengths and limitations of our analyses and model in terms of volcano monitoring and surveillance.

### Physical interpretation of CFc unrest

The interpretation of the current prolonged unrest of CFc is a matter of open debate^28,32,108^. Various theories have been proposed and tested either on the bradyseism of 1983–1984 and on the mini-uplifts^62^, as well as on the decennial-like accelerating uplift after 2005. For instance, Macedonio et al.^79^ hypothesized that the intrusion of sills can be responsible for some of the dynamics observed during CFc unrest, and developed a dynamic model of sill intrusion in a shallow volcanic environment^50,64,65^. Similarly, Amoruso et al.^2,3^ interpreted the deformation history of the CFc in 1980–2010 and 2011–2013 as a consequence of paired deformation sources, i.e. a quasi-horizontal crack at a depth of about 3600 m in the central part of the caldera, and a small spheroid located at about 1900 m depth under Solfatara. Moretti et al.^83^ hypothesized that shallow sills, intruded during 1969–1984, have completely cooled, so that the ongoing uplift is mostly led by deeper, CO_2_-richer, magmatic gases. Finally, De Siena et al.^58^ studied the seismic data during the 1983–1984 unrest episode, constraining a 4–4.5 km deep aseismic zone of high attenuation offshore Pozzuoli, a 3–4 km deep reservoir of supercritical fluids under Pozzuoli, and a shallower aseismic deformation source under Solfatara. Many studies found that geochemical signals on many occasions followed geophysical signals by a few months, or they were simultaneous^38,42,107,109^, and a systematic geochemical monitoring was carried out at Solfatara and Pisciarelli since the 1980s^33,45,80,81^. A mechanism of repeated volcanic or hydrothermal system pressurization that culminates in injection of fluids along a conduit-like path, which allows fluids discharge and temporary depressurization of the source region was also recently theorized^43,66^. Then, analyzing recent episodes of seismicity and gas emission that occurred in 2015 and 2019, a shallow conduit-like seismogenic structure in Pisciarelli zone was identified^68^.

In this context, our data analyses are able to describe the current CF unrest as the superimposition of two clearly distinct trends: i) a slowly accelerating trend with decennial-like time scale and ii) a cyclical, although characterized by several periods, alternation of speed-ups and slow downs, including the mini-uplifts and their seismic trace.

With regards to the decennial-like trend, several previous studies discussed the ongoing slowly accelerating inflation of CFc, and empirically described the monitoring signals by both hyperbolic and exponential growth curves^41,74,84^. We note that the FFM analysis also adopts hyperbolic functions, which grow faster than exponential functions. As said above, the reason of such decennial-like uplift since 2005 is debated as generated between repeated magma injections and sill expansion, or large gas fluxes^64,65,111,113^. However, regardless the nature of its forcing source, the prolonged accumulation of stress and crustal damage increase the possibility of reaching critical pre-eruptive conditions^75^.

The present analysis shows that, since 2015, the rate of seismicity, both in terms of event count and strain release, speeds up faster than the rate of ground displacement. This is different from what had been observed before 2015, i.e. seismicity increasing at about the same rate of the ground uplift and of the concentration of the fumarolic gas species more sensitive to temperature^39,40,42^. The interpretation of such decoupling between the trends of deformation and seismicity is challenging. However, it may be related to the approaching of a transition from a quasi-elastic brittle regime to inelastic regime of deformation, in which the number of seismic events is expected to accelerate faster as the proportion of inelastic deformation becomes larger^74,75^. In fact, in a completely inelastic regime all deformation is associated to a pervasive rupture of materials and irreversible deformation.

As regards the cyclical trends with different periods, we found that the cyclical component of the dynamics is present, with consistent properties, both in the phase of subsidence before 2005 and in the phase of uplift since 2005; moreover, it is shared by both the ground displacement and the seismic data, and it is synchronous between them. Regardless of their uncertain interpretation, the recognition and quantification of these unrest cycles represent significant information in terms of forecasting the likely evolution of the unrest signals, not less than their decennial-like accelerating trend.

The interpretation of the measured unrest cycles is also not trivial. They do not appear to be strictly seasonal effects, as they do not express a precise annual recurrence. Although the driving mechanism of these cycles could plausibly be in the periodic deep inflow of the magmatic fluids, this is not necessarily the case. The driving mechanism of these oscillations remains unclear and we cannot ultimately exclude an exogenous climate-related origin^61,92,99^. For instance, rainfall data of the CFc from 2000 to 2020 had several oscillations, both seasonal and over multiple years. Examples of these data are reported in Supporting Information S9. While the longest of these cycles may concur to drive the oscillations that we observe in the CFc monitoring signals, the timing of climate-related oscillations does not precisely match with the geophysical data, at least indicating a complex interaction with the hydrothermal and volcanic systems.

In addition, the theory of poro-elastic media may explain the amplification or self-generation of waves of pressure, especially when the input of deep fluids is slowly but steadily increasing^108^. In this conceptual model, first an increased pressure from a deep source or a temporary reduction in the hydrothermal permeability drives more fluids in a shallow reservoir than those that it is able to discharge. This raises the pressure in the reservoir, both slowing down the ingression of new fluids from depth, and eventually increasing the extrusion of fluids to the surface, thus generating a temporary speed-up of the ground displacement and seismicity^68^. Then, the extrusion of fluids reduces the pressure in the reservoir, allowing a new input wave of fluids thus favoring a new wave of pressure. The characteristic time of these phenomena, and the observation of secondary oscillations, would likely depend on the geometry of the shallow reservoir and could explain the observed signals’ variations, together with the possible climate-related effects mentioned above. Self-sealing of the porous medium in which deep fluids are injected, caused by salt precipitation in the porous matrix, was also invoked as a possible reason for the recurrent cycles till 2016^113^.

### Operational application of the data analysis

In this study we did not analyze the statistics of past eruptions of CFc and their recurrence times^12–14^, but instead focused on the mathematical features of geophysical monitoring signals. More specifically, this study aims at producing quantitative estimates of the prolonged increase, transient evolution, and temporary peaks in the geophysical monitoring signals’ rates observed at CFc, rather than eruption forecasting sensu stricto. In fact, despite the approach of an eruption would be very likely associated with a substantial acceleration in the unrest signals, this condition is not per se sufficient. For instance, and this is common for calderas, variations in the dynamics of the shallow hydrothermal system can cause temporarily accelerating signals. Similarly, “arrested” (or failed) eruptions are also possible, in which the volcano displays the precursory symptoms typical of an impending eruption, but does not culminate with magma reaching the surface^17^.

However, the quantitative analyses of the data can provide useful insights into the dynamics of the volcanic system and its evolution. For instance, the rate peaks clearly mark times of accelerated dynamics and possible state transition. When the background seismicity rate drops dramatically after a maximum, that marks a transition between a time of stress release by microseismicity, and a time of relatively quiescent stress build up. Similarly, an anomalous pressurization of the hydrothermal system could lead to dangerous consequences regardless of its source^97,98^. Possible eruptive and not-eruptive phenomena can unexpectedly occur after elusive precursor signals, hence motivating the usefulness of any near real-time tool that allow an improved tracking of the system evolution^5^. In addition, we emphasize that the monitoring signals are not smooth functions and typically possess multi-scale properties, i.e. the same data can be analyzed at various time scales (e.g. days, weeks, months, years) highlighting different dynamics likely corresponding to different physical mechanisms and possible outcomes^34,35,44,55,56,105^.

A remaining challenge is whether monitoring signals can distinguish between pre-eruptive and non-eruptive outcomes, and how deformation and seismic rates could accelerate to bulk failure^70,75,114^. With this respect, the application of the FFM to the Campi Flegrei crisis, with all the assumptions and limits that we explained above, highlights that a possible continuation of the acceleration observed in the last about 15 years implies that a potential critical state of the volcano could not be excluded in the future, also providing a temporal estimate in which this eventuality may occur. This is useful information to volcano observatories responsible for assessing the evolution of a volcanic crisis, and, ultimately, to civil protection authorities and civil society^76,96^.

In fact, the FFM waiting time index reported in Fig. 11 is a quantitative indicator of the periods of most intense acceleration of the monitoring signals, and could help anticipating the climax of the phases of increased unrest also by constraining their possible duration in case of continuation of the observed trends. However, it is worth clarifying that the reported forecasts are not absolute probabilities of occurrence of eruptive events due to the extremely simplified nature of the model adopted and since the observed decennial-like accelerating trends of data could change in the future, either slowing down or accelerating further.

## Conclusions

In this study, we analyzed the instrumental catalogs of ground displacement and earthquakes in CFc, continuously collected in the last two decades (2000–2020), and compared them with data collected in 1983–1999. We measured and described the main features of the monitoring signals variations, in terms of temporal rates and rate changes, Fourier spectrum and differential modeling of their nonlinear acceleration.

We summarize the main findings of the data analysis as follows (a more analytical summary is in Supporting Information S10).

- Geophysical monitoring data show two clearly overlying trends: i) a slow, i.e. decennial-like, acceleration and ii) cyclical oscillations at various frequencies. These two trends have dissimilar time scales and sum up to produce the observed signals in the last decades.
- The slowly accelerating trend started at least since 2005, and 90–97% of the increase of the signals has occurred since 2011, 40–80% since 2018. Since 2011, the GPS and seismic signals did not follow the same trends, and the seismic count increased faster than the ground displacement, and even more clearly since 2015. The ground displacement shows a linearized rate slope, i.e. acceleration, of 0.6 cm/yr^2^ and 0.3 cm/yr^2^ from 2000 to 2020 respectively for the vertical (RITE GPS) and the horizontal (ACAE GPS) components. The seismic count and the strain release have a nonlinear accelerating trend growing faster than an exponential function, possibly like a hyperbolic function.
- From 2000 to 2020 there have been seven main rate maxima, one oscillation every 3.2 ± 0.4 years. In addition, Fourier analysis shows a longer cycle at 6.5–9 years, which could be obtained by considering the most evident peaks only. We also observed secondary maxima at integer fractions of 1/2, 1/3, 1/4 of the main inter-maxima. The leveling data collected at benchmark 25A display four additional maxima with similar properties in the period 1985–2000.
- We used past data to make a retrospective analyses by applying the FFM and its stochastic formulation (pFFM) able to consider several sources of uncertainty affecting the system. Various regression lengths, i.e. 10-year, 5-years, 3-years, enabled us to study either the decennial-like accelerating trend or the cyclical oscillations. The failure forecast method applied on 01/01/2021 provides an upper limit for the possible future continuation of the slowly accelerating trend, e.g. [0, 3, 11] years if applied to the seismic count (excl. swarms) from 2010 to 2020, [0, 6, 21] years if based on the GPS data (ACAE GPS, horizontal displacement). Retrospective analysis of the method also identified a sequence of four main transient phases of increased acceleration, in 2006–2007, 2011–2013, 2016–2017, 2019–2020.

The identification and characterization of the decennial-like accelerating trend that is recorded since 2005 in the investigated geophysical data is probably the most relevant and critical result emerged from this study. In particular, the application of the FFM approach indicates that, in case of continuation of this trend in the coming years, and under all the model assumptions and limitations above described, a critical state of the volcano could not be excluded in the future, also providing a first temporal estimate of this possibility. Moreover, a prosecution of such long-term trend could amplify the effects of new periodic oscillations that characterized the dynamics of the volcano since 1985 due to an increased stress level and ground rock damage.

We believe the results here presented are useful information to better characterize the current status of CFc as well as to forecast its possible future behavior. At the time of writing this manuscript (October 2022) the trends that we described are continuing with similar characteristics, in particular the RITE uplift has reached 99 cm since 2005, which has exceeded the previous maximum level of 1985 (INGV periodic bulletin of Campi Flegrei; http://www.ov.ingv.it).

## Supplementary Information

Supplementary Information.

## Data Availability

The dataset S1 generated during the current study is available in the repository https://doi.org/10.5281/zenodo.6807713. The seismic catalogs of CFc are stored in the INGV—Osservatorio Vesuviano web site https://www.ov.ingv.it/index.php/cataloghi-sismici-vulcani-napoletani (last accessed 11 October 2022). All other data generated or analyzed during this study are included in this article (and its Supplementary Information files).
