# Supplementary material for: Data analysis of the unsteadily accelerating GPS and seismic records at Campi Flegrei caldera from 2000 to 2020
Source: Sci Rep. 2022 Nov 10;12:19175. doi: 10.1038/s41598-022-23628-5 (PMC9649781; doi:10.1038/s41598-022-23628-5)
Supplement: Supplementary file 1 — Supplementary Information. [file 41598_2022_23628_MOESM1_ESM.pdf]

Supporting Information for

Data analysis of the unsteadily accelerating GPS and seismic records at Campi Flegrei caldera from 2000 to 2021.

Andrea Bevilacqua(1), Prospero De Martino(2), Flora Giudicepietro(2), Patrizia Ricciolino(2), Abani Patra(3), E. Bruce Pitman(4), Marcus Bursik(5), Barry Voight(6), Franco Flandoli(7), Giovanni Macedonio(2), Augusto Neri(1)

[1]Istituto Nazionale di Geofisica e Vulcanologia, Sezione di Pisa, Pisa, Italy
[2]Istituto Nazionale di Geofisica e Vulcanologia, Osservatorio Vesuviano, Napoli, Italia
[3]Dpt. of Mathematics, Tufts University, Medford, MA, USA
[4]Dpt. of Materials Design and Innovation, University at Buffalo, Buffalo, NY, USA
[5]Dpt. of Earth Sciences, University at Buffalo, Buffalo, NY, USA
[6]Dpt. of Geology, Pennsylvania State University, State College, PA.
[7]Scuola Normale Superiore di Pisa, Pisa, Italy

Additional Supporting Information

Captions for Dataset S1
Permanently deposited into https://doi.org/10.5281/zenodo.6807713.

Contents of this file

Figure S2 to S9
Text S10

Data Set S1. Extended dataset of all the analyses

This compressed folder contains supporting information related to the Figures in the manuscript.
Files and folders labeled with G1...n are related to the GPS data, those labeled with H1...n are related to the seismic data.

In particular:
Subfolder 1_DATA supports Figure 3 – the vertical and the horizontal components of ground displacement at all analyzed GPS stations; the logarithmic plots of all seismic events and of their energy. It also shows the complete plot leveling data from 1905 to 2010 (modified from del Gaudio et al., 2010). It also includes Figure 2 and Figure 6a-c.

Subfolder 2_AnnualRate supports Figure 4 - the annual rate of the vertical and horizontal components of ground displacement at all analyzed GPS stations; the annual rate of all seismic events and of their energy. These detail the 2-year, the 6-month, and the 30-day average results. It also supports Figure 5 with similar data concerning 2018-2020.

Subfolder 3_InverseRate supports Figure S3 - the inverse rate of the vertical and the horizontal components of ground displacement at all analyzed GPS stations; the inverse rate of all seismic events and of their energy. These detail the 2-year, 6-month, and 30-day average results, including detailed plots of 2018-2020.

Subfolder 4_RateChange supports Figure S2 - the daily rate change of the vertical and horizontal components of ground displacement at all analyzed GPS stations; the daily rate change of all seismic events and of their energy. These detail the 2-year, the 6-month, and the 30-day average results, including detailed plots of 2018-2020.

Subfolder 5_FourierCoef supports Figure 6 - the Fourier spectrum of the vertical and the horizontal components of ground displacement at all analyzed GPS stations. These detail the 2-year, 6-month, and 30-day average results obtained in 2000-2020, 2011-2020, 2018-2020. Also, additional plots that detail other combinations of time domain and part of the Fourier spectrum, thus testing the sensitivity of the main harmonics on the time domain selected.

Subfolder 6_ FFM_WaitTime supports Figure 11 – waiting time examples based on vertical and horizontal components of ground displacement at all analyzed GPS stations; all seismic events, and their energy. These detail the 2-year, 6-month, and 30-day average rate results, and the 10-year, 5-year and 3-year regressions.

Subfolder 7_FFM_FailTime also supports Figure 11 – all the results expressed in terms of the failure time t_f instead of in terms of the waiting time [$t_f(t)$ - t].

Subfolder 8_pFFM_Regression supports Figure 9 - the pFFM examples based on the vertical and the horizontal components of ground displacement at all analyzed GPS stations; all seismic events and of their energy. These detail the 2-year, 6-month, and 30-day average rate results, and the 10-year, 5-year and 3-year regression.

Subfolder 9_pFFM_Probability supports Figure S4 - pFFM examples based on vertical and horizontal components of ground displacement at all analyzed GPS stations; all seismic events, and their energy. These detail the 2-year, 6-month, and 30-day average rates, and the 10-year, 5-year and 3-year regressions.

Subfolder 10_BarplotProb supports Figure S5 - results expressed in terms of the mean failure time probability at 2, 5, 10, and 25 years.It also supports Figure S6 - examples based on 6-month, and 30-day average rate results.

Subfolder 11_BarplotWaitTime supports Figure S6 - all the results expressed in terms of the waiting time (t_f – t) barplot. It also includes Figure S5.

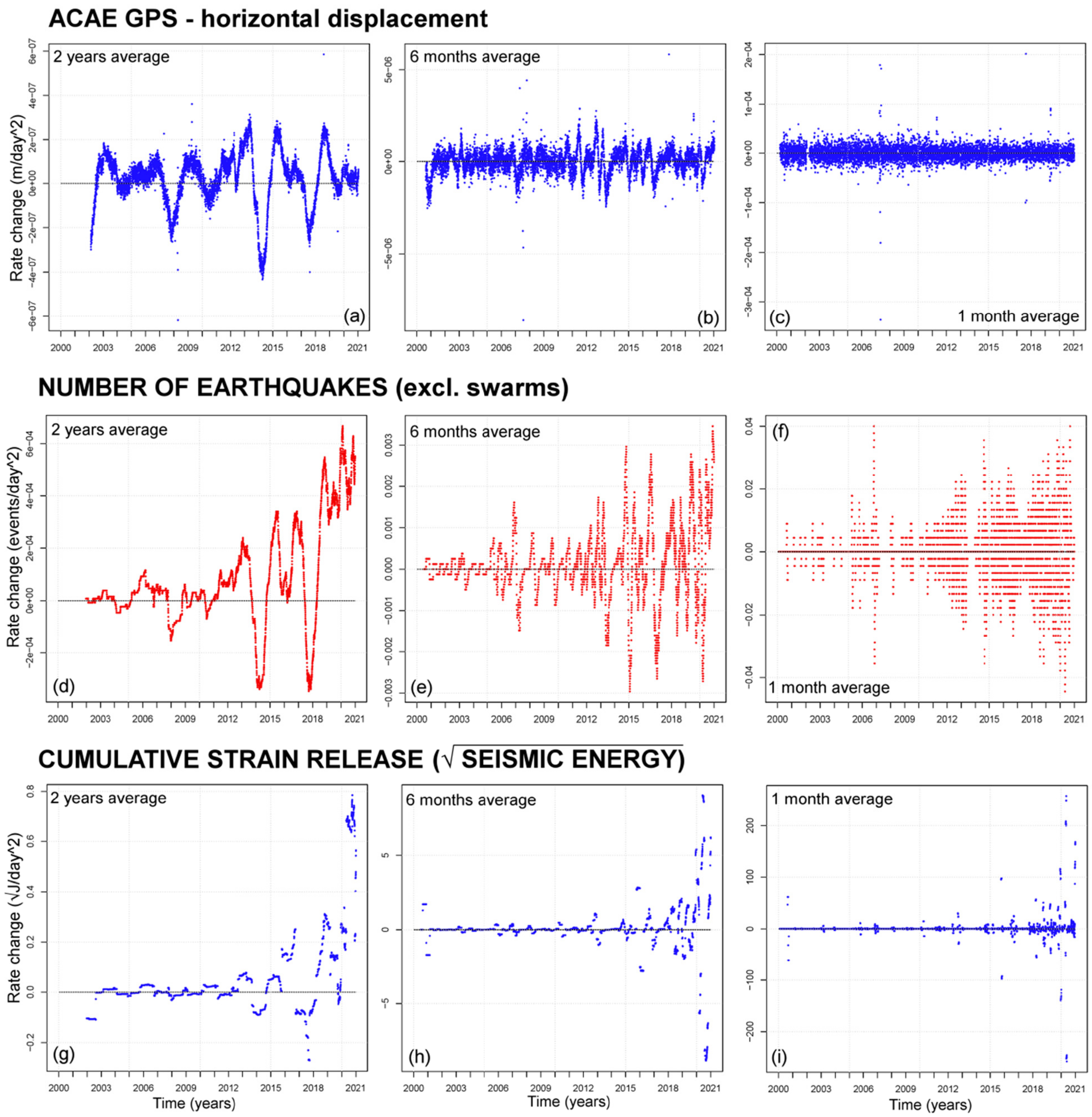

Figure S2. Rate change (second derivative)

Addendum to Figure 4

Examples of daily rate change graphs. Plots (a-c) show the ground displacement rate change; plots (d-f) show the rate change of seismic events recorded (excl. swarms); plots (g-i) show the seismic strain rate change estimation. Plots (a,d,g) are calculated on a 2-year moving average, plot (b,e,g) on a 6-month moving average, plot (c,f,i) on a 30-day moving average. Supporting information S1 extends these results.

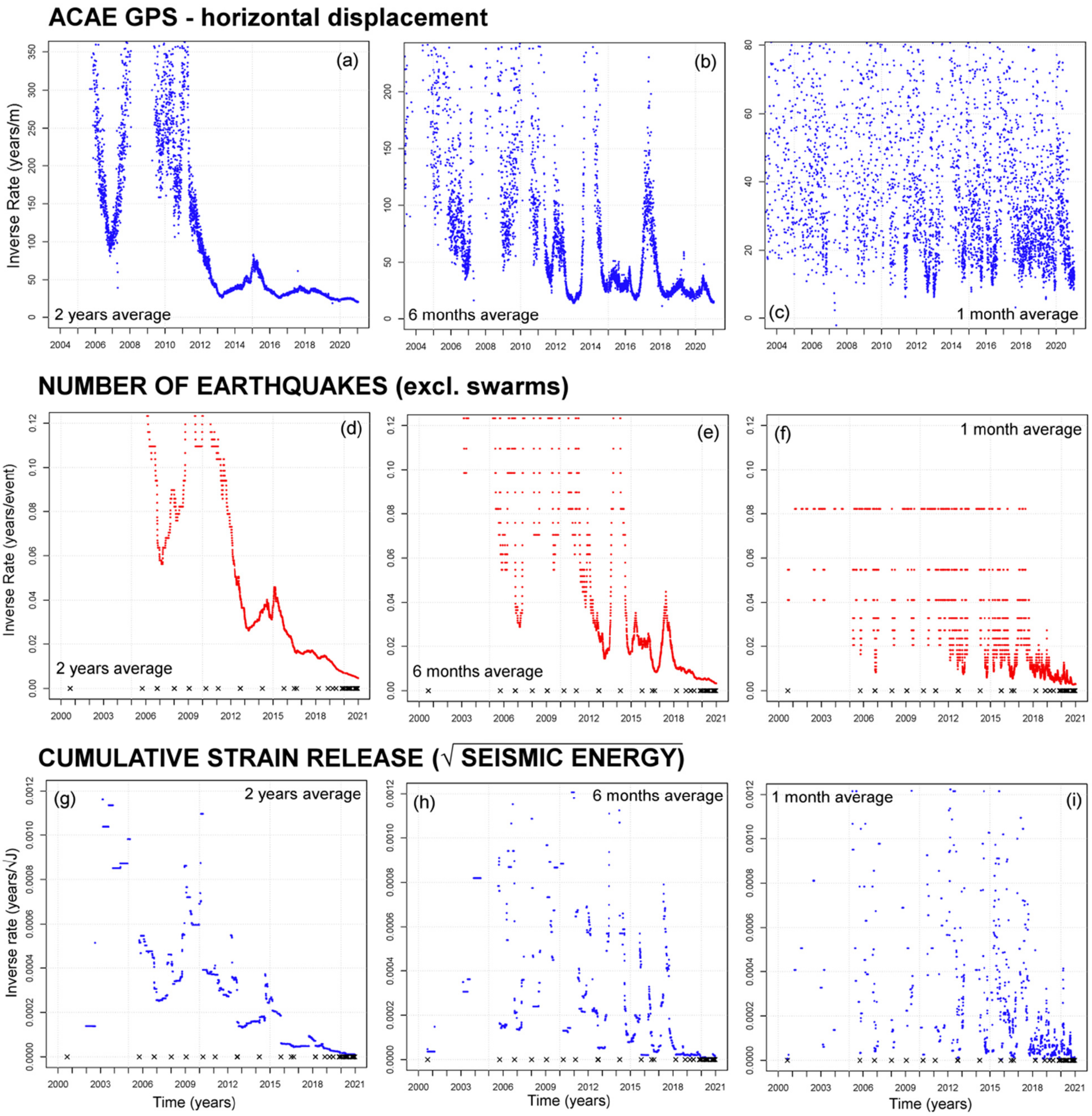

Figure S3. Inverse rates

Addendum to Figure 9

Examples of inverse rate graphs. Plots (a-c) show the ground displacement inverse rate; plots (d-f) show inverse rate of seismic events recorded (excl. swarms); plots (g-i) show seismic strain inverse rate estimation. Plots (a,d,g) are calculated on a 2-year moving average, plot (b,e,g) on a 6-month moving average, plot (c,f,i) on a 30-day moving average. The swarms of > 20 events are marked with black crosses in (d-i). Supporting information S1 extends these results.

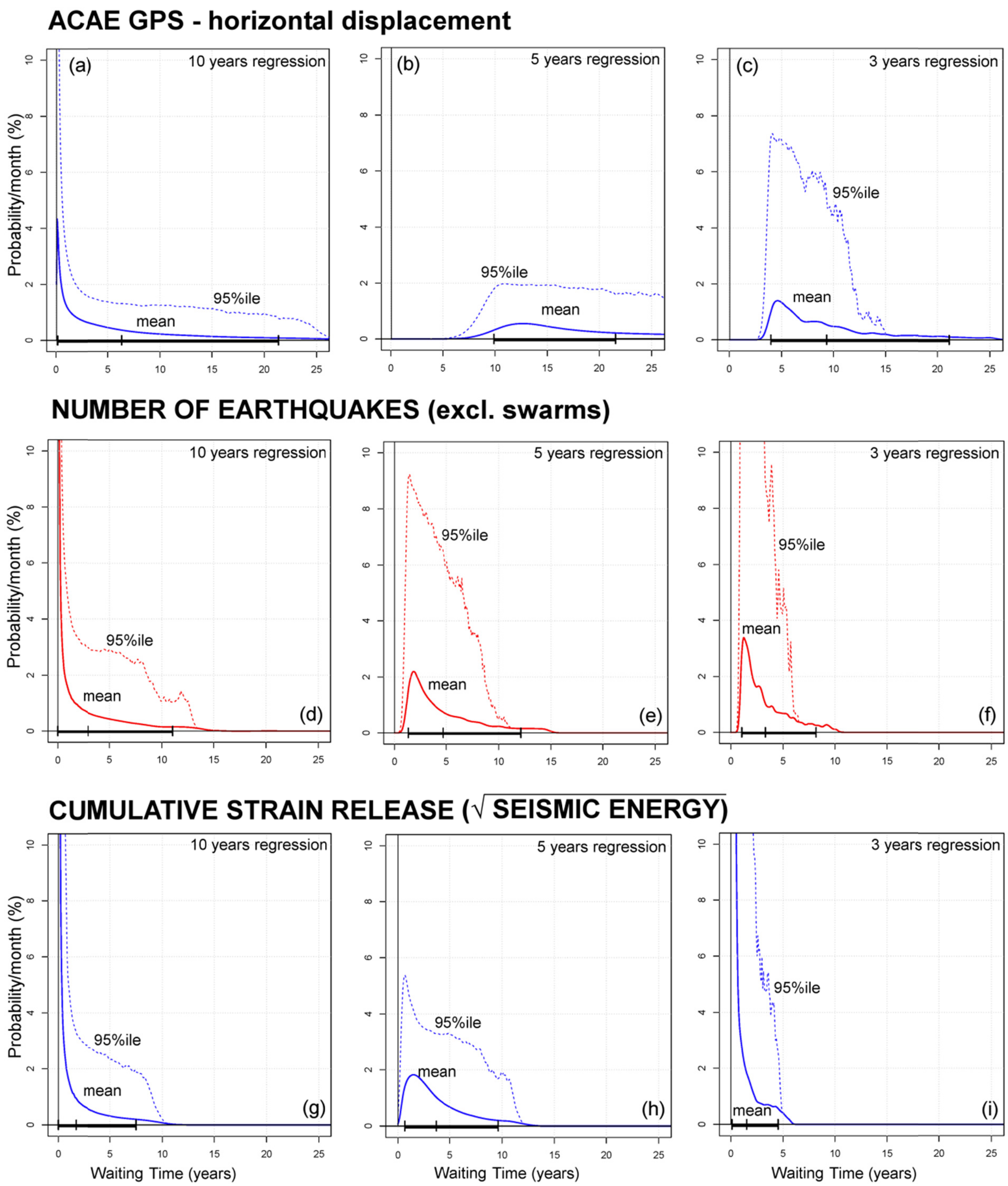

Figure S4. Failure time probability density functions
Addednum to Figure 9

Examples of pFFM probability density functions. The forecast is formulated at t = 01/01/2021, marked by a vertical line. The failure time t_f is expressed in terms of the waiting time (t_f - t). A solid colored curve shows the mean t_f PDF and a dashed line mark its upper 95th percentile. The 5th percentiles of the PDF values are negligible. A bold interval on the x-axis marks the mean and the 90% confidence of the pFFM forecast. Plots (a,d,g) use a 10-year regression, plots (b,e,h) a 5-year regression, plots (c,f,i) a 3-year regression. Plots (a-c) are based on the inverse rate of ground displacement; plots (d-f) on the seismic events recorded (excl. swarms); plots (g-i) on the seismic strain estimation. All inverse rates are calculated on a 2-year moving average. Supporting information S1 extends these results.

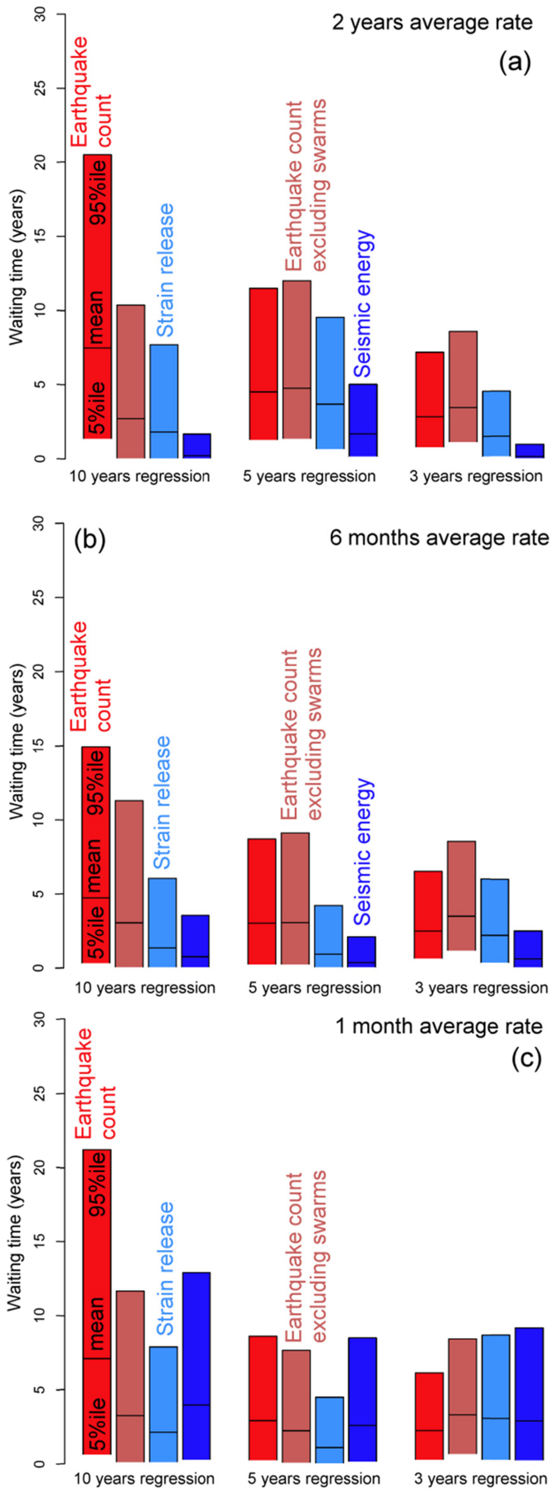

Figure S5. Comparison of waiting time based on seismic data (1 Jan 2021)
Addendum to Figure 10

Barplot of the waiting time obtained with the pFFM applied to seismic count and energy data. The forecast is formulated at t = 01/01/2021. The bars show the mean and the 90% confidence of the pFFM forecast. Different data are marked with different colors: the count of all earthquakes in the catalog is red, the count excluding swarms is brown, strain release is light blue, seismic energy is blue. Examples are based on 10-year (left), 5-year (center), and 3-year (right) regressions. Plot (a) uses a 2 years moving average, plot (b) a 6-month moving average, plot (c) a 30-day moving average. Supporting information S1 extends these results.

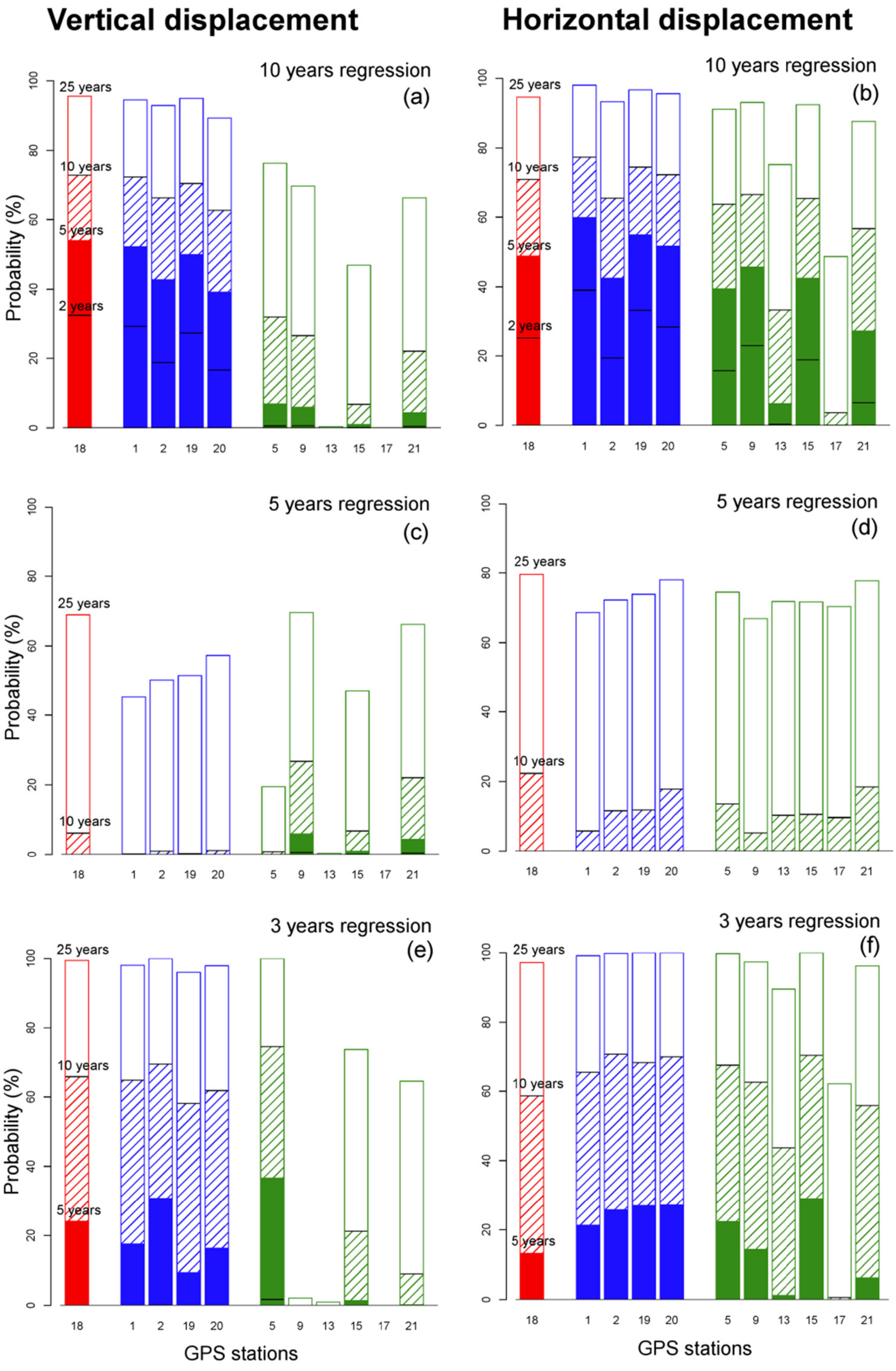

Figure S6. Spatial comparison of failure time probability based on GPS data (1 Jan 2021) Addendum to Figure 10

Barplot of the failure time probability obtained with the pFFM and applied to the ground displacement data. The forecast is formulated at t = 01/01/2021. Plots (a,c,e) are based on the inverse rate of vertical ground displacement; plots (b,d,f) on the horizontal displacement. The bars show the probability levels at 2 (black mark), 5 (solid color), 10 (colored stripes) and 25 years (white) from t. The eleven analyzed stations are marked with different colors: RITE station is red, four proximal stations are blue, six distal stations are green. Plots (a,b) use a 10-year regression, plots (c,d) a 5-year regression, plots (e,f) a 3- year regression. All inverse rates are calculated on a 2-year moving average. Supporting information S1 extends these results.

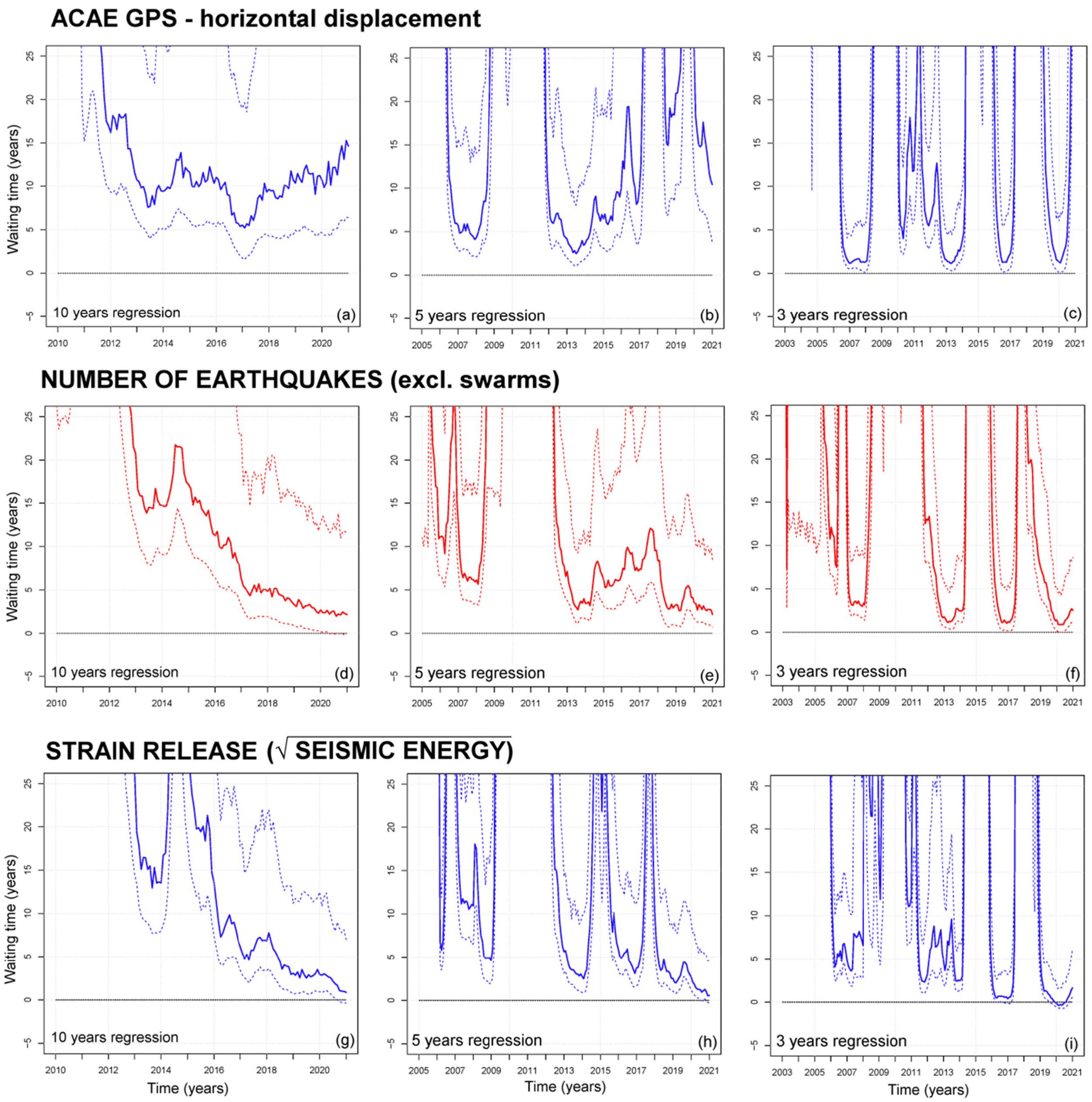

Figure S7. Retrospective analysis of the waiting time from 2000 to 2020, 6-month average rate Addendum to Figure 11

The failure time t_f is expressed in terms of the waiting time (t_f - t). The bold colored line is the mean forecast and the dashed lines are the 5th and 95th percentiles of its uncertainty range. Plots (a,d,g) use a 10-year regression, plots (b,e,h) a 5-year regression, plots (c,f,i) a 3-year regression. Plots (a-c) are based on the inverse rate of ACAE GPS ground displacement; plots (d-f) on seismic events recorded (excluding swarms); plots (g-i) on seismic strain estimation. All inverse rates are calculated on a 6-month moving average. Supporting information S1 extends these results.

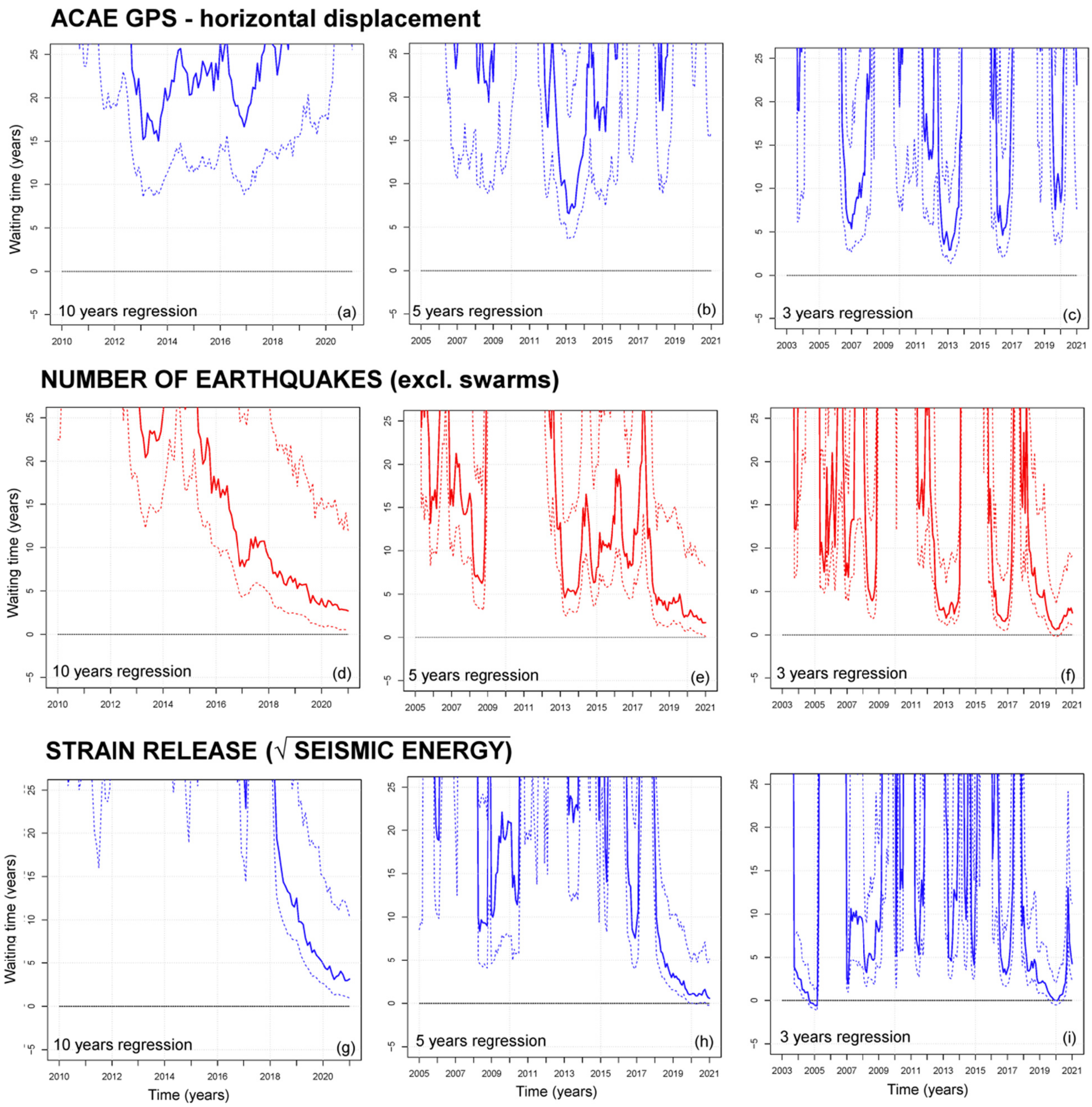

Figure S8. Retrospective analysis of the waiting time from 2000 to 2020, 30-day average rate Addendum to Figure 11

The failure time t_f is expressed in terms of the waiting time (t_f - t). The bold colored line is the mean forecast and the dashed lines are the 5th and 95th percentiles of its uncertainty range. Plots (a,d,g) use a 10-year regression, plots (b,e,h) a 5-year regression, plots (c,f,i) a 3-year regression. Plots (a-c) are based on the inverse rate of ACAE GPS ground displacement; plots (d-f) on seismic events recorded (excluding swarms); plots (g-i) on seismic strain estimation. All inverse rates are calculated on a 30-day moving average. Supporting information S1 extends these results.

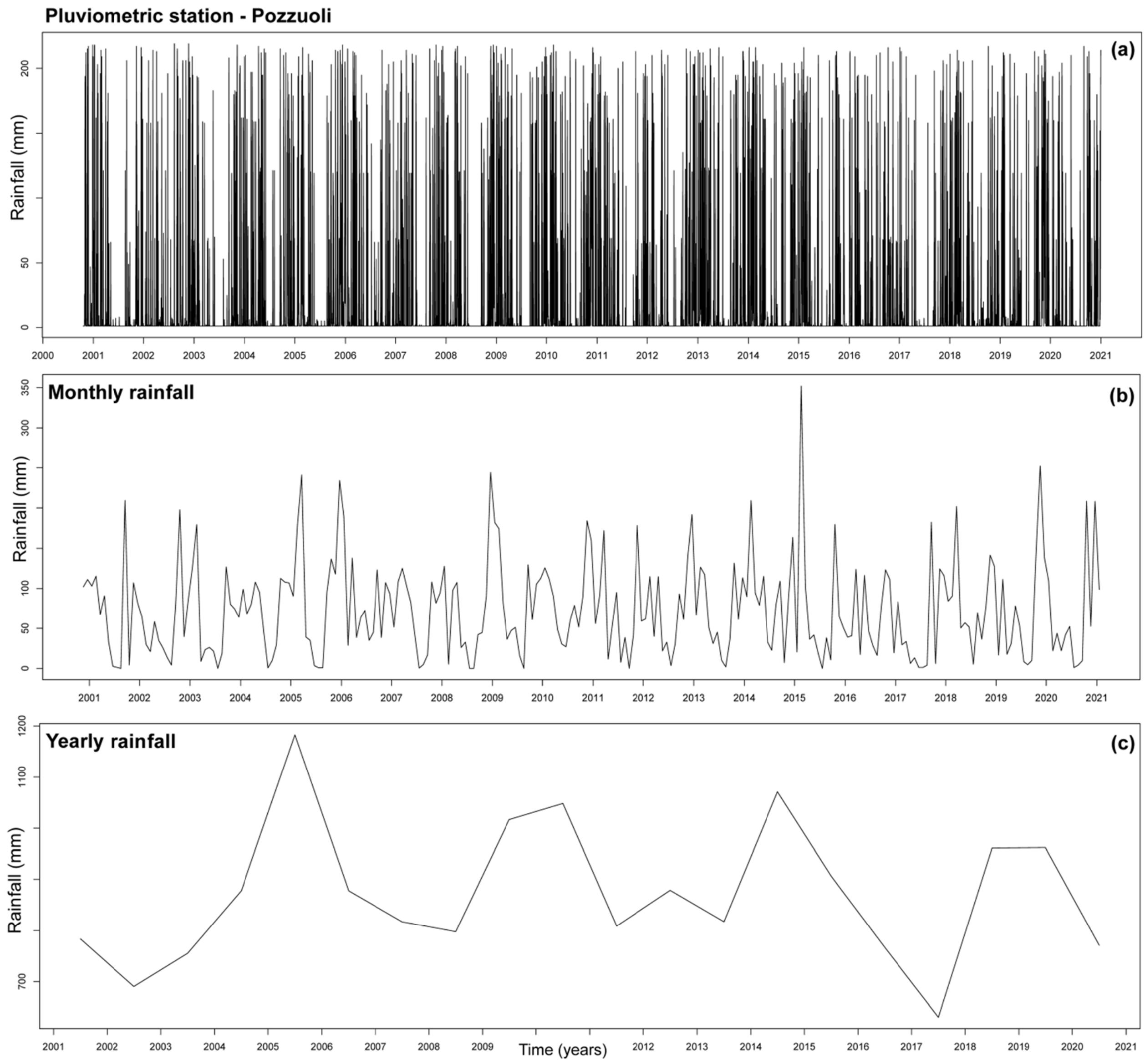

Figure S9. Rain fall analysis from 2000 to 2020

Examples of pluviometer data recorded at Pozzuoli station, near the center of the CFc. The plots show the (a) daily, (b) monthly, (c) yearly rain fall. Dataset published by Civil Protection of Regione Campania, Italy. (http://centrofunzionale.regione.campania.it/#/pages/sensori/archivio-pluviometrici)

Text S10. Detailed summary of results

The three strands of data, i.e. horizontal displacement, seismic count without swarms, and seismic strain, are all accelerating, at least since 2005, but they differently evolved over time, especially since 2015.

- A vertical displacement of ~ 78 cm (RITE GPS) and a horizontal displacement of ~ 34 cm (ACAE GPS) has occurred since 2005, of which ~ 90% has occurred since 2011 and 40% since 2018. The total seismic count excluding swarms is ~ 900 events, of which ~ 90% has occurred since 2011, 60% since 2018. Total seismic energy release amounts to 3 GJ since 2000, with > 97% being released since 2015, 80% since 2018.
- Before 2011, the horizontal displacement increased erratically for the mini-uplifts, and the seismic count was concave-shaped in log-scale. Since 2011, both the horizontal displacement and seismic count have almost been linear in log-scale, but the former graph was concave-shaped, the latter became convex-shaped, thus growing faster than an exponential function, possibly like a hyperbolic function. The seismic strain has always been convex-shaped in log-scale.

The annual rate of the data is significantly rough on short time scales, and thus notable changes depend on the time scale selected for averaging, i.e. 2-year, 6-month, and 30-day.

- The horizontal displacement at ACAE GPS from 2000 to 2020 produces a linearized rate slope, i.e. acceleration, of 0.3 cm/yr^2 regardless of the time step selected. The linearized fit of the vertical displacement at RITE GPS station is 0.6 cm/yr^2 and shows similar features to the horizontal rate. In addition, an alternation of speed-ups and slow-downs occur at various frequencies.
- The annual rate of seismic count excluding swarms reached from 210 to 350 events/year in 2020, depending on the time scale of the average rate. More swarms occurred from 2018 to 2020 than from 2000 to 2017, i.e. 20 swarms vs 14. There was an alternation of speed-ups and slow-downs and the rate trend has been nonlinearly accelerating.
- The seismic strain release surpassed 10000 √J/year on a 2-year average. Concentrated peaks corresponding to the strongest earthquakes intermittently but significantly contribute to the total strain release.

The rate change of the three strands of data shows an alternation of positive and negative values, but with several differences.

- The horizontal displacement is overwhelmed by noise in the 30-day average. The plots lack of significant increasing trend and are suitable for Fourier analysis.
- The seismic count excluding swarms produces waves with amplitude that becomes larger with time. The 2-year rate change has accelerated almost linearly since 2011, and has always been positive since 2018.
- The seismic strain release produces waves with amplitude that becomes significantly larger with time. The 2-year average rate change has always been nonlinearly accelerating.

We described in closer detail the annual rate of the data from 2018 to 2020 and from 1983 to 1985.

- In 2018-2020, the 6-months average rates of both the ground displacement data and the seismic count excluding swarms display an alternation of three speed-ups and slow-downs, with period of about 1.6 years between the two maxima at 03/2019 and 12/2019. Plus, variable waves with periods from 3 to 8 months are evident in both the 30-day average rate and the 6-months rate change of the seismic count.
- In 1983-1985, the bradyseismic crisis included three great swarms, the first and the third including > 250 events, and the second > 400 events. The annual rate of the number of earthquakes, their strain release and seismic energy, averaged at 30-day time steps, display five peaks at 07/1983, 10/1983, 04/1984, 09/1984, 12/1984. The intervals between the peaks are ~ 3 months for the first and the fourth, and ~ 5 months between the middle peaks.

A synoptic panel shows that alternation of minima and maxima in the GPS and seismic data are significantly consistent. Thus, the Fourier analysis of the GPS data can describe both.

- Synchronous records of seismic count and the ground displacement display seven maxima: in 2000, 2003, 2006, 2009, 2013, 2016, 2019. The inter-maxima times are 2.8, 3.4, 2.8, 3.5, 3.4, 3.3 years,

i.e. 3.2±0.4 years. The 6-month average rate highlights a number of secondary peaks between each of the main peaks; the secondary inter-maxima have integer proportions of 1/2, 1/3, 1/4 of the main inter-maxima.

- The leveling data collected at benchmark 25A in 1985-2010 display additional maxima in 1985, 1989, 1994, 1996/97, 2000. The inter-maxima times are 4.5, 4.9, 2.5, 3.7 years; i.e. the first and second intervals were 40-50% longer than average.
- The Fourier analysis of 2-year average rate produces a peak Fourier coefficient at 3.2 years and a wide interval of high coefficients, from 6.5 to 9 year-periods. The Fourier analysis of 6-month average rate shows a main peak at 1.65 years. The Fourier analysis of 30-day average rate describes less regular recurrence for the shortest harmonics – peaks at 2.9, 3.6, 4, and 5.7 months appear in the data of the different GPS stations.

Through the analysis of the inverse rate we applied the FFM to the GPS and seismic data on 1 Jan 2021.

- The 2-year average inverse rate of the horizontal displacement displays local minima in 2006-2007, 2013-2014, and then a plateau since 2016-2017. The 6-month and especially the 30-day average detail a greater number of local minima and gaps. In the inverse rates of seismic count and strain release the plateau since 2016-2017 is absent, and becomes an overall decrease.
- The horizontal displacement produces failure times at [0, 6, 21] years, [10, 21, >25] years, [4, 9, 21] years, respectively, based on 10-year, 5-year, and 3-year regressions. The monthly PDFs show maximum values of 2-10% and prolonged values at 0.5-1% for several years after the peak, with uncertainty ranges up to 1.5%, 2% and 5% respectively.
- The seismic count produces failure times at [0, 3, 11] years, [1, 5, 12] years, [1, 3, 8] years, respectively - more steady forecasts than in the ground displacement case. The monthly PDF has slightly greater maxima and prolonged values than the GPS, but also greater uncertainty ranges. The strain release produces failure times at [0, 1, 7] years, [0, 4, 10] years, [0, 1, 5] years, respectively, i.e. the shortest obtained.

The retrospective analysis of the FFM from 2000 to 2020, describes the temporal evolution of the forecasts.

- Based on a 10-year regression the waiting times decrease in mean value after 2012, except for the GPS data during 2020, and the strain release in 2014-2017. In 2012-2017 the mean values based on GPS are smaller than those based on seismic data, while after 2018 it is the opposite.
- Based on 5-year and 3-year regression the data indicate four transient phases of reduced waiting times – in the latter case they are 2006-2007, 2011-2013, 2016-2017, 2019-2020. In the GPS data the first phase ends earlier, and all the gaps are more evident. In 2018-2020 the waiting times based on GPS are greater, especially in 2020.

The comparison of the eleven GPS stations reveals the spatial sensitivity of the FFM algorithm, which is most significantly affected by the regression length.

- The waiting times based on a 10-year regression are similar in RITE and in the four GPS proximal stations, with failure time probability of 40-60% in 5 years, and 65-75% in 10 years, on average. Instead, the 6 distal stations produce smaller or more uncertain estimates.
- Based on a 5-year regression of vertical displacement the failure time probability in 10 years is ~6% in RITE, and 0-1% in the proximal stations. For the horizontal displacement, the failure time probabilities in 10 are ~22% in all the stations, on average. Based on a 3-year regression the probability estimates increase again in the proximal stations.

The comparison of seismic data shows that the FFM results have a greater sensibility on the regression length than on the time step in the signals' rate calculation, and the type of data.

- The waiting time estimates mostly decrease from seismic count to strain release to seismic energy, except for the 30-day average rates, where seismic energy is consistent with seismic count.
